# The age of violence: Mapping brain age in psychosis and psychopathy

**DOI:** 10.1016/j.nicl.2022.103181

**Published:** 2022-09-06

**Authors:** Natalia Tesli, Christina Bell, Gabriela Hjell, Thomas Fischer-Vieler, Ivan I Maximov, Genevieve Richard, Martin Tesli, Ingrid Melle, Ole A Andreassen, Ingrid Agartz, Lars T Westlye, Christine Friestad, Unn K Haukvik, Jaroslav Rokicki

**Affiliations:** a Norwegian Centre for Mental Disorders Research (NORMENT), Institute of Clinical Medicine, University of Oslo, Oslo, Norway; b Norwegian Centre for Mental Disorders Research (NORMENT), Division of Mental Health and Addiction, Oslo University Hospital, Oslo, Norway; c Department of Psychiatry, Oslo University Hospital, Oslo, Norway; d Department of Psychiatry, Østfold Hospital Trust, Graalum, Norway; e Division of Mental Health and Addiction, Vestre Viken Hospital Trust, Drammen, Norway; f Department of Health and Functioning, Western Norway University of Applied Sciences, Bergen, Norway; g Department of Psychology, University of Oslo, Oslo, Norway; h Department of Mental Disorders, Norwegian Institute of Public Health, Oslo, Norway; i Department of Adult Psychiatry, Institute of Clinical Medicine, University of Oslo, Norway; j Department of Psychiatric Research, Diakonhjemmet Hospital, Oslo, Norway; k Centre for Psychiatry Research, Department of Clinical Neuroscience, Karolinska Institutet & Stockholm Health Care Services, Stockholm County Council, Stockholm, Sweden; l Centre of Research and Education in Forensic Psychiatry, Oslo University Hospital, Oslo, Norway; m University College of Norwegian Correctional Service, Oslo, Norway

**Keywords:** Psychosis, Brain age, Antisocial behaviour, Forensic psychiatry, Neuroimaging

## Abstract

Young chronological age is one of the strongest predictors for antisocial behaviour in the general population and for violent offending in individuals with psychotic disorders. An individual's age can be predicted with high accuracy using neuroimaging and machine-learning. The deviation between predicted and chronological age, i.e., brain age gap (BAG) has been suggested to reflect brain health, likely relating partly to neurodevelopmental and aging-related processes and specific disease mechanisms. Higher BAG has been demonstrated in patients with psychotic disorders. However, little is known about the brain-age in violent offenders with psychosis and the possible associations with psychopathy traits.

We estimated brain-age in 782 male individuals using T1-weighted MRI scans. Three machine learning models (random forest, extreme gradient boosting with and without hyper parameter tuning) were first trained and tested on healthy controls (HC, n = 586). The obtained BAGs were compared between HC and age matched violent offenders with psychosis (PSY-V, n = 38), violent offenders without psychosis (NPV, n = 20) and non-violent psychosis patients (PSY-NV, n = 138). We ran additional comparisons between BAG of PSY-V and PSY-NV and associations with Positive and Negative Syndrome Scale (PANSS) total score as a measure of psychosis symptoms. Psychopathy traits in the violence groups were assessed with Psychopathy Checklist-revised (PCL-R) and investigated for associations with BAG.

We found significantly higher BAG in PSY-V compared with HC (4.9 years, Cohen's *d* = 0.87) and in PSY-NV compared with HC (2.7 years, *d* = 0.41). Total PCL-R scores were negatively associated with BAG in the violence groups (*d* = 1.17, *p* < 0.05). Additionally, there was a positive association between psychosis symptoms and BAG in the psychosis groups (*d* = 1.12, *p* < 0.05).

While the significant BAG differences related to psychosis and not violence suggest larger BAG for psychosis, the negative associations between BAG and psychopathy suggest a complex interplay with psychopathy traits. This proof-of-concept application of brain age prediction in severe mental disorders with a history of violence and psychopathy traits should be tested and replicated in larger samples.

## Highlights

• Young age is one of the strongest predictors for antisocial behaviour.
• Brain age gap (BAG) is the deviation between predicted and chronological age.
• We found higher BAG in violent offenders with psychosis compared with controls.
• We found no differences in BAG between violent and non-violent psychosis subjects.

## Graphical abstract.

**Figure:**
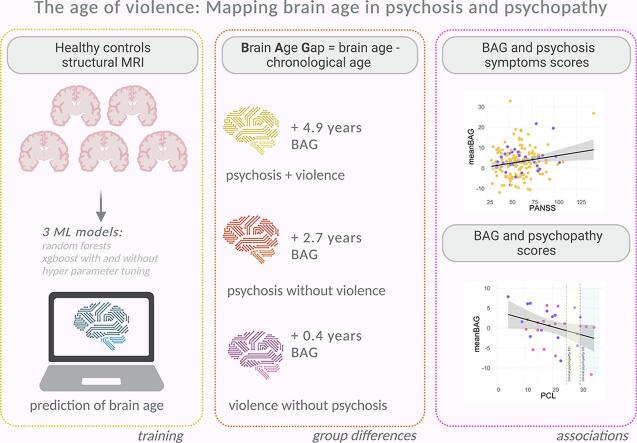
Graphical abstract was created using BioRender.com

## 1 Introduction

Violence and antisocial behaviour in individuals with psychotic disorders challenge clinical practice and optimal treatment, with devastating consequences for the persons involved, their families and society at large. Epidemiological and clinical research has identified several risk factors related to violent behaviour in psychotic disorders, including substance abuse, positive psychotic symptoms, previous criminal history and male sex (Fazel et al., 2017, Witt et al., 2013). Further, the presence of psychopathy traits heightens violence risk (Laajasalo et al., 2011), with a 4-fold increase in likelihood for violent recidivism in schizophrenia patients with comorbid psychopathy (Tengstrom et al., 2000).

In the general population, the association between age and violence is well-established (Rocque et al., 2015), with the peak for incarceration for any offense, including violent crime, occurring during early adolescence (<25 years) and gradually declining with age. This phenomenon, known as *the age-crime curve* is one of the most consistent and well-documented observations in developmental criminology (Farrington, 1986, Moffitt, 1993, Moffitt, 2018, Shulman et al., 2013). Further, age has been identified as one of the strongest predictors for estimating risk of violent crime in severe mental illness (https://oxrisk.com/oxmiv/), with a decreasing likelihood of violence with increasing age (adjusted odds ratio 0.63 per 10 years of age in a cohort aged 15–65 years) (Fazel et al., 2017).

The age-dependency of violent behaviour has been suggested to be partly explained by the neurobiological processes underlying improvements in decision making, impulse control and emotion regulation which occur during brain development beyond early adolescence (Arain et al., 2013). In general, structural maturation of the human brain is underpinned by complex processes involving both progressive and regressive changes characterised by regional specificity (Storsve et al., 2014). It has been shown that adolescents exhibiting antisocial behaviour (conduct problems, callous-unemotional and psychopathic features) have increased grey matter volumes (De Brito et al., 2009) and cortical thickness (Yang et al., 2015) in regions subserving cognitive and emotional regulatory functions compared with their peers. While these findings have been suggested to indicate a delay in brain maturation (Blair, 2009), antisocial behaviour and psychopathic traits in adult individuals have been generally associated with grey matter reductions in prefrontal, limbic and paralimbic regions (Boccardi et al., 2011, Ermer et al., 2012, Johanson et al., 2019, Yang et al., 2010), thus indicating a complex interplay between the phenotypic life course trajectories and structural brain development.

Accordingly, it has been demonstrated that chronological age may not be able to capture the overall neurocognitive, physiological and disease-specific aging processes (Franke et al., 2013, Gaser et al., 2013). Recent advances in neuroimaging and machine learning techniques have enabled accurate prediction of age at an individual level (Cole and Franke, 2017). Interestingly, application of brain age measures proved to outperform chronological age in prediction of recidivism in a sample of incarcerated male offenders (Kiehl et al., 2018), thus indicating that proxies based on structural brain age may be better suited to account for individual differences in biological ageing than the chronological one. But little is known about deviation between the predicted and chronological age, referred to as brain age gap (BAG) in relation to violence and psychosis. BAG has been suggested to reflect both the general characteristics of brain health, likely relating to neurodevelopment and aging as well as specific disease-related neurodegenerative mechanisms (Cole et al., 2019, Franke and Gaser, 2019).

In schizophrenia, the relation between chronological and brain-predicted age has been extensively investigated, with studies reporting higher BAG using structural (Kaufmann et al., 2019, Koutsouleris et al., 2014, Nenadic et al., 2017), blood perfusion (Rokicki et al., 2021) and diffusion weighted imaging (Tonnesen et al., 2020) data. Increased brain-age in this patient group may indicate reduced residual lifespan and converges with recent reports from epidemiological studies showing that males with psychotic disorders lose at least 10 life-years compared with males in the general population, largely due to natural causes such as cardiovascular and other non-communicable diseases (Plana-Ripoll et al., 2019, Tesli et al., 2022). While a few explorative studies in schizophrenia patients with a history of violence applied machine learning based on clinical and sociodemographic predictors (Kirchebner et al., 2020, Sonnweber et al., 2021) and neuroimaging data (Gou et al., 2021), there are no previous studies of brain-age prediction in this patient subgroup. Indeed, we lack knowledge on the interplay between the apparent brain aging in psychosis, history of violence, and psychopathy traits. Further, we need more insight into how psychopathy traits map onto brain maturation patterns in individuals with and without psychotic disorders.

Here we present the first proof-of-principle application of brain-age prediction in psychosis, violent behaviour, and psychopathy traits. Specifically, we aimed at mapping brain deviations based on BAG-defined measures derived from brain morphology using T1-weighted structural MRI in violent offenders with and without psychosis and their associations with psychopathy features. Based on previous studies, we hypothesised that psychotic disorders would be associated with a higher BAG compared with healthy controls. Due to mixed neurostructural findings in psychopathy, we remained agnostic about the expected direction of effects for the associations between BAG and groups with a history of violence and psychopathy.

## 2 Materials and methods

### 2.1 Participants

The final sample consisted of 782 male participants. All participants were recruited from the greater Oslo region as part of four studies: the Thematically Organized Psychosis study (TOP), the STROKEMRI study, The Youth TOP study (uTOP), and The Forensic Psychiatry study (sTOP). The inclusion restricted exclusively to male individuals was due to very low number of recruited female subjects in the study (1 in the violent offenders with psychosis group), thus precluding investigation of putative sex differences in brain age or associations with history of violence.

The inclusion criteria for participants in the sTOP study (violent offenders with psychosis (PSY-V), and non-psychotic violent offenders (NPV)) were age between 18 and 70 years, absence of head trauma leading to loss of consciousness and no current or previous somatic illness that might have affected brain morphology. The NPV group consisted of incarcerated persons serving a preventive detention sentence, which is the most severe sanction according to the Norwegian penal law and is imposed in cases of particularly serious crimes involving interpersonal violence.

The inclusion criteria for participants in the TOP study (non-violent psychosis group, PSY-NV) were following: a diagnosis of psychosis spectrum disorder based on the DSM-IV criteria, age 18–65 years. Healthy control subjects (HC) were randomly selected from the Norwegian national population registry (http://www.ssb.no/en). The HC were screened with the Primary Care Evaluation of Mental Disorders to confirm no history of psychiatric disorder. Younger participants (age 12–18 years) were included from the uTOP study. The inclusion criteria for these participants were similar to the TOP study, a psychosis diagnosis was based on DSM-IV criteria using the Norwegian version of the Schedule for Affective Disorders and Schizophrenia for School Aged Children (6–18 years) present and lifetime version (Kaufman et al., 1997). The inclusion criteria for the HC from the STROKEMRI study were age at or above 18 and no history of neurological or psychiatric disorder.

The study was approved by the Norwegian Regional Committee for Medical Research Ethics. All participants and their guardians provided written informed consent to participate in the study. Key demographics are described in detail in Table 1 and supplementary Fig. S1. Key clinical information is summarised in Table 2. Detailed inclusion criteria are described in the supplementary material M1.

**Table 1:** Participant demographics summarised by diagnosis. Abbreviations: HC – healthy controls, NPV – violent offenders without psychosis, PSY-NV – non-violent psychosis patients, PSY-V – violent offenders with psychosis, *N* – number of participants, *SD* – standard deviation. Data quality was estimated using MRIqc random forest classifier (scale from 0 to 1, the smaller the number, the better the image quality).

Group	N	Age, mean (years)	Age, SD (years)	Age, min (years)	Age max, (years)	Data quality, mean	Data quality, SD
HC	586	39.7	16.0	12.7	92.0	0.401	0.151
NPV	20	42.4	14.4	22.7	71.0	0.403	0.137
PSY-NV	138	29.0	8.7	15.1	57.8	0.416	0.133
PSY-V	38	34.7	8.9	19.2	54.1	0.461	0.151
Total	782	37.7	15.2	12.7	92.0	0.407	0.148

**Table 2:** Mean, standard deviation (sd) and 1st and 3rd quantiles (Q) of interquartile range of IQ and clinical scores of incarcerated participants and patients with psychosis. As scores were available for a subset of subjects with imaging data, the sample size (n) is smaller than the total sample size of respective groups. Abbreviations: NPV – violent offenders without psychosis; PSY-V – violent offenders with psychosis; PSY-NV – non-violent psychosis patients, NA – not applicable.

	HC	NPV	PSY-NV	PSY-V
	Wechsler Abbreviated Scale of Intelligence (IQ)			
*n*	413	17	128	19
*mean*	114.0	101.6	102.4	93.8
*sd*	10.6	12.8	14.4	15.1
*Q1*	109.0	90.0	92.0	85.5
*Q3*	121.0	109.0	113.0	108.0

	Global Assessment of Functioning (symptoms, GAF-S)			
*n*	0	0	134	34
*mean*	NA	NA	48.1	41.6
*sd*	NA	NA	13.7	10.7
*Q1*	NA	NA	39.0	35.0
*Q3*	NA	NA	58.8	48.8

	Global Assessment of Functioning (function, GAF-F)			
*n*	0	0	131.0	34.0
*mean*	NA	NA	48.1	38.9
*sd*	NA	NA	13.2	7.6
*Q1*	NA	NA	39.5	35.0
*Q3*	NA	NA	55.5	44.8

	Positive and Negative Syndrome Scale (PANSS)			
*n*	0	20	137	34
*mean*	NA	39.4	60.8	64.6
*sd*	NA	11.2	17.1	18.9
*Q1*	NA	33.0	50.0	51.0
*Q3*	NA	39.25	70.0	80.8

	Defined daily dose (DDD) for antipsychotic medication			
*n*	0	0	123	34
*mean*	NA	NA	1.06	1.58
*sd*	NA	NA	0.83	0.79
*Q1*	NA	NA	0.60	0.96
*Q3*	NA	NA	1.33	2.06

### 2.2 Clinical assessments

The assessment of violence for PSY-V and NPV was based on court files and hospital records. The inclusion criteria for these two groups were murder, attempted murder as well as severe physical assaults towards other people (including sexual assaults) according to the MacArthur criteria (Monahan et al., 2000). Both groups were institutionalised at the time of inclusion in the study due to perpetration of violent crime; the PSY-V group was hospitalised at high security psychiatric wards and the NPV group was incarcerated at high security prisons.

Psychopathy traits were evaluated with the Psychopathy Checklist-revised (PCL-R) (Hare, 2003). The PCL-R applies a 20-item scale to measure behavioural patterns and personality traits associated with the construct of psychopathy both in research and forensic settings. The evaluation procedure was based on an in-depth interview as well as inspection of the individual’s history of violent offending including court documentation and/or medical records. The PCL-R assessment was performed by certified psychiatrists and psychologists calibrated through the official PCL-R training. The same two raters scored both psychotic and non-psychotic offenders to ensure internal consistency of evaluations in the prisons and hospital wards.

To ensure no previous history of violence in the nonviolent psychosis group, their medical files have been thoroughly examined. This procedure entailed evaluation of all study inclusion protocols, which are based on detailed information obtained from medical records including both structured interview with the patient and clinical journals.

Current psychosis symptoms were rated with the Positive and Negative Syndrome Scale (PANSS) in both psychosis groups (PSY-V and PSY-NV) as well as in NPV group (Kay et al., 1987). IQ was measured in all groups with the Norwegian version of the Wechsler Abbreviated Scale of Intelligence (WASI-II) (Hays et al., 2002) by trained psychologists.

### 2.3 MRI acquisition, image quality control and image pre-processing

T1-weighted volumes were collected on two 3 T scanners (GE Medical Systems and DiscoveryTM (MR750)) located at the Oslo University hospital, Norway. (1) GE Signa HDxt scanner with a standard 8-channel head coil, using a sagittal 3D fast spoiled gradient echo (FSPGR) sequence with the following parameters: repetition time (TR) = 7.8 ms, echo time (TE) = 2.9 ms, flip angle 12°, slice thickness 1.2 mm, 166 sagittal slices, field of view (FOV) 256 mm × 256 mm, acquisition matrix 256 × 192 mm, voxel size = 1 × 1 × 1.2 mm^3^ and (2) DiscoveryTM (MR750) scanner with the vendor's 32-channel head coil, using an inversion recovery-fast spoiled gradient echo sequence (BRAVO) with the following parameters: TR = 8.16 ms, TE = 3.18 ms, TI = 450 ms, flip angle = 12°, FOV = 256 mm, acquisition matrix = 256 × 256 mm, 188 sagittal slices, slice thickness = 1.0 mm, voxel size = 1 × 1 × 1 mm^3^.

### 2.4 Image quality control

Image quality control was performed as a two-step process. First, all T1w images were processed with MRIQC (Esteban et al., 2019). The images classified by a default machine learning algorithm to an exclude node with a probability of at least 0.5 were further visually investigated by two trained raters (NT and JR). In the second step, quality assessment of the area and thickness of cortical maps was performed by a careful visual inspection of lateral and medial snapshots of all maps by the same raters. The participant was excluded if the surface values included negative values, uncharacteristic patterns or strong value disbalance between hemispheres.

### 2.5 Image pre-processing

Briefly, *FreeSurfer v7.1 (*Fischl, 2012) was used to extract 34 cortical thickness and area region of interest (ROI) values, in addition to average thickness and total area in each hemisphere based on the Desikan-Killiany atlas (Desikan et al., 2006). The choice of this particular atlas was based on its high intra and inter reliability with manual ROI parcellation (Iscan et al., 2015), its common use in large projects such as UK Biobank and ABCD-study as well as in research on brain age estimation (Rokicki et al., 2021, Vidal-Pineiro et al., 2021), thus providing transparency and facilitating replication of our findings. We extracted volumes of 45 subcortical ROIs based on the automated volume segmentation (Fischl, 2012). Additionally, 30 hippocampal subfield and amygdala nuclei volumes per hemisphere (60 in total) were obtained by applying the hippocampal subfield segmentation algorithm as provided in *FreeSurfer* (Iglesias et al., 2015). All area and volume ROI values were controlled for total intracranial volume (ICV) by building a linear model in the training set. To harmonise data from different scanners we used neuroCombat R package (Fortin et al., 2018) (Supplementary Figs. S2-S4). ComBat calculates scanner-specific location as well as scale parameters for each feature independently and subsequently pools information across features using empirical Bayes, thus improving parameter estimation, particularly for small sample size studies (Fortin et al., 2017).

### 2.6 Brain age gap calculation

For brain age prediction we used a feature set based on cortical thickness, area and subcortical volumes, in total n = 245 features per individual (full list is provided in the supplementary material M2). We divided data into a training and testing set, full workflow is presented in Supplementary Fig. S5. The testing set consisted of patients and HC matched for age and scanner (Ho et al., 2011).

To train the models described below we used data obtained from 390 male HC. Next, we tested the performance of our trained models by predicting age in unseen matched HC in the test sample (196 individuals). More specifically, we calculated the Spearman’s correlation between the predicted and the chronological age before age bias removal, as well as the root mean square (RMSE) and mean absolute error (MAE) in years.

To account for the small sample size and to reduce the likelihood of spurious findings, we used three different models to evaluate BAG: random forest, extreme gradient boosting with and without parameter optimisation:

1. Random forest algorithm as implemented in randomForest package in R (Breiman, 2001), which is known for its resilience to overfitting, robustness to noise and few hyperparameters to tune. To determine the optimal value of predictors sampled for splitting at each node, we used tuneRF function from the same library. We grew 5,000 trees, as more trees provide more robust and stable error estimates and variable importance measures (Boehnke, 2019).
2. Extreme gradient boosting, an ensemble model based on gradient tree boosting (Chen and Guestrin, 2016); trained in an additive manner with sequential addition of learners so that prediction error from previous model estimates is corrected. This model was implemented in XGBoost package in R, an algorithm and has been shown to accurately predict brain age in in a recent large scale age prediction study (Kaufmann et al., 2019) with the following parameters: learning rate eta = 0.1, nround = 5000, gamma = 1, max_depth = 6, subsample = 0.5 (defaults).
3. To improve the model even further we used extreme gradient boosting with hyper parameter tuning by applying random search procedure as implemented in mlr R package.

**Fig. 1:**
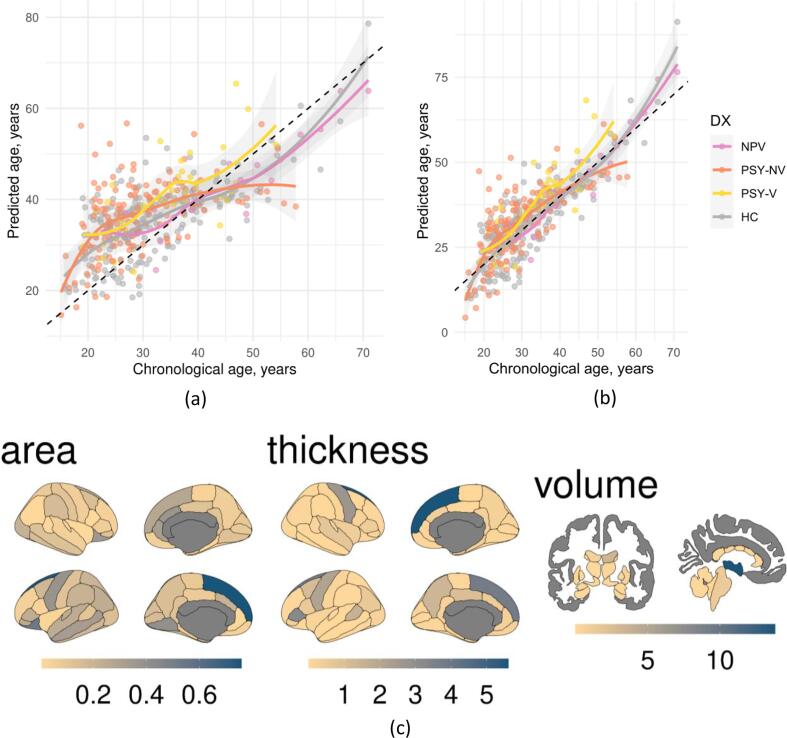
Association between chronological and predicted age before (a) and after (b) controlling for brain age bias. Color points and lines represent different participant groups. Dashed black line represents an ideal fit. Brain map of feature contribution measured as gain for brain age prediction (c). Based on the xgboost model with standard parameters.

We adjusted predicted brain age for the brain prediction bias using the method proposed in (Beheshti et al., 2019). First, we calculated slope and intercept of linear regression between the predicted brain age gap and chronological age, and then we subtracted these from predicted age and added chronological age. The slope and intercept were calculated in the training set and then applied to left out patients and age matched healthy controls (Fig. 1ab and Supplementary Fig. S6).

To estimate the features contributing the most to brain age prediction we used the mean increase in mean squared error (MSE) for random forest and gain for XGBoost models. MSE quantifies the difference between randomly shuffled and actual values for the investigated feature, while keeping the rest of the features intact when applied on unseen data. Gain represents the fractional contribution of each feature to the model based on the total gain of this feature's splits. Higher percentage means a more important predictive feature. To visualise the feature importance, the results were mapped onto segmented brain surface using the ggseg R package (Mowinckel and Vidal-Piñeiro, 2020).

### 2.7 Statistical analyses

In order to assess group differences between patients and HC, we matched subjects to controls with respect to age using nearest neighbour matching with 1:1 ratio and logistic regression distance as implemented in the R package *matchIt* (Ho et al., 2011). The subsequent between group statistical analyses were performed using general linear models (GLM) as implemented in the permutation analysis of linear models (PALM) (Winkler et al., 2014) toolbox with 10,000 permutations while controlling for effects of age, and demeaning the data including covariates in the design matrix. We used family wise error correction to correct for the number of contrasts (2) and number of models (3). Additionally, we corrected for the number of groups with FDR correction (four groups: HC vs PSY-V, PSY-NV, NPV and PSY-NV vs PSY-V). The results were corrected for 24 tests in total. Additionally, to assess effect sizes we calculated Cohen's *d*. To calculate the BAG in years between two groups we calculated the difference between the means of the underlying distributions.

In our supplementary analyses we ran pairwise comparisons for the groups with a history of violence (NPV and PSY-V) with age-matched HC while controlling for the diagnosis of psychosis in the statistical model. Additionally, the two psychosis groups (PSY-V and PSY-NV) were compared with HC while controlling for violence in the statistical model.

To assess associations between BAG averaged over three brain age prediction models and PCL-R, PANSS, antipsychotic medication, calculated as defined daily dose (DDD), and IQ we used GLM in the PALM toolbox with 10,000 permutations with PCL-R, PANSS total score, DDD of antipsychotic medication, and IQ entered as predictors and controlling for the effects of age and group in four separate models.

## 3 Results

### 3.1 Predicting brain age in healthy controls

The best age prediction performance was achieved by XGBoost with standard parameters model MAE = 6.6 (*r*^2^ = 0.46), followed by XGBoost with parameters optimisation MAE = 7.1 (*r*^2^ = 0.43), and random forest MAE = 7.3 (*r*^2^ = 0.45). The complete list of fit results is presented in Table 3. The models showed high agreement in age prediction with correlation of 0.91 and above both for HC and patients (the full list of correlations is listed in the Supplementary Table T1).

**Table 3:** Model fit results in out of sample HC (*n* = 196). MAE – mean absolute error, RMSE – root mean square error, rMAE and rRMSE errors after controlling for brain age bias.

Model	*r*^2^	MAE, years	RMSE, years	rMAE, years	rRMSE, years
*RF*	0.45	7.3	8.9	4.6	5.8
*XGB-std*	0.46	6.6	8.3	5.3	6.8
*XGB-opt*	0.43	7.1	8.7	5.2	6.4

In general, feature importance patterns were in agreement, as Pearsońs correlation (Supplementary Fig. S7) over all feature importance metrics ranged from 0.89 to 0.97 (all *p_corr_* < 0.05, Bonferroni adjusted for 12 comparisons, 4 modalities × 3 models). Volume based features showed highest correlation (*r* = 0.94–1.00 all *p_corr_* < 0.05) and area-based lowest, yet still significant (*r* = 0.44–0.73, all *p_corr_* < 0.05). Further, out of top 10 predictive features, 6 were in common across 3 models (Supplementary Tables T2-T4).

The top 3 features contributing most to age prediction in the XGBoost with standard and optimal parameters coincided and included white matter hypointensities volume, 3rd ventricle volume and right superior frontal thickness (Fig. 1c). For random forest the top 3 features were: left pallidum, mean thickness of right hemisphere and left superior frontal thickness (Supplementary Fig. S8). For the most precise XGBoost model with standard parameters among top 20 features: 8 were volume, 11 were thickness and 1 was area. 3 features were bilateral, 8 from right and 9 from left hemisphere. The lists with top 20 contributing features are presented in the Supplementary Tables T2-T4.

### 3.2 Group differences in BAG

**Fig. 2:**
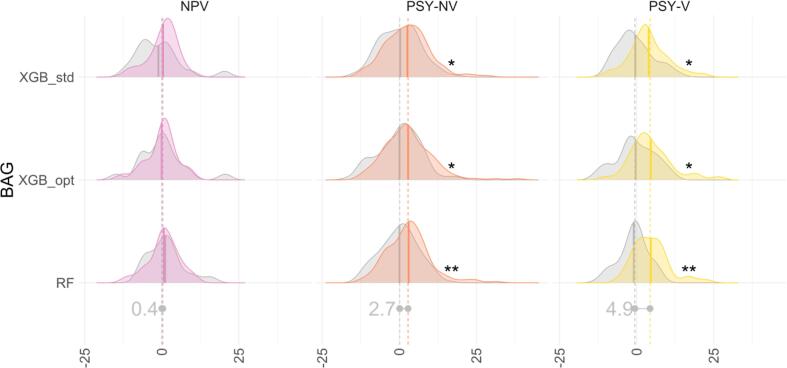
Group comparison of BAG in HC vs NPV, PSY-NV PSY-V groups. Both distributions and means are shown. Asterisks on the right side indicate significant results (FDR corrected), with *p* <.05, *p* <.01 and *p* <.001 being marked as 1 to 3 asterisks, respectively. Distributions for HC are shown in grey. Abbreviations: HC – healthy controls, NPV – violent offenders without psychosis, PSY-NV – non-violent psychosis patients, PSY-V – violent offenders with psychosis.

Demographics for each group comparison are listed in the Supplementary Table T5. The results are shown in Fig. 2, Table 4 and Supplementary Fig. S6.

**Table 4:** Mean group differences between NPV, PSY-NV and PSY-V and age and sex matched HC. Abbreviations: HC – healthy controls, NPV – violent offenders without psychosis, PSY-NV – non-violent psychosis patients, PSY-V – violent offenders with psychosis, Cd – Cohen’s *d*. BAG was additionally controlled for age within each comparison, Cd, *t*-values and p-values were calculated with PALM using permutation modelling. *p*-values were adjusted for 3 models (FWE) × 2 contrasts (FWE) × 4 groups (FDR) = 24 comparisons.

	BAG diff, years	Cd	t_value_	p_adjusted_	
	HC/NPV (n = 20/20)				
*RF*	−0.42	−0.08	−0.25	1.00e + 00	
*XGB_std*	1.57	0.27	0.85	7.26e-01	
*XGB_opt*	0.05	0.01	0.02	1.00e + 00	

	HC/PSY-NV (n = 138/138)				
*RF*	2.91	0.47	3.69	6.60e-03	**
*XGB_std*	2.34	0.37	2.86	1.82e-02	*
*XGB_opt*	2.74	0.39	3.07	1.25e-02	*

	HC/PSY-V (n = 38/38)				
*RF*	5.56	1.11	4.68	1.20e-03	**
*XGB_std*	4.36	0.74	3.15	1.25e-02	*
*XGB_opt*	4.88	0.75	3.19	1.25e-02	*

	PSY-NV/PSY-V (n = 38/38)				
*RF*	1.61	0.28	1.19	5.81e-01	
*XGB_std*	1.55	0.26	1.08	5.81e-01	
*XGB_opt*	1.70	0.26	1.08	5.81e-01	

All models showed significantly higher BAG in both psychosis groups as compared to HC, with the PSY-V group having largest effect (4.9 years, *p* <.05, Cohen's *d* = 0.87), followed by PSY-NV (2.7 years, *p* <.05, *d* = 0.41). The PSY-V had higher BAG, though the difference was not significant when compared to PSY-NV (1.6 years, *p* >.05, *d* = 0.27). Additionally, the NPV had higher BAG, yet again not significant when compared to HC (0.4 years, *p >.05*, *d* = 0.07).

**Fig. 3:**
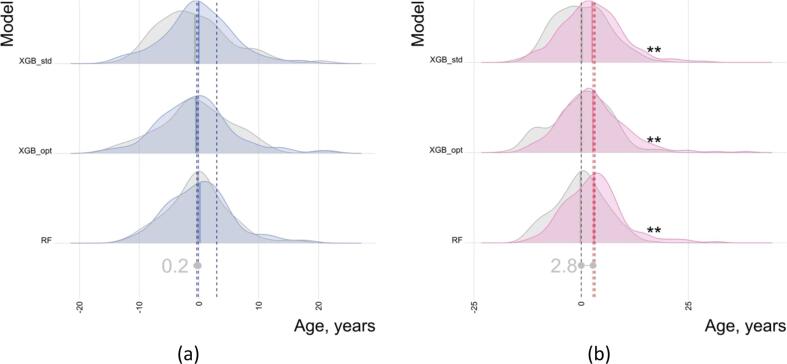
Group comparison of BAG in HC vs violence controlled for psychosis (a) and psychosis controlled for violence (b). Both distributions and means are shown. The right most lines indicated by the dark blue (a) and dark red (b) show means without controlling for psychosis (a) and violence (b). Asterisks on the right side indicate significant results (multiple comparisons corrected), with *p* <.001 being marked as 3 asterisks. Distributions for HC are shown in gray. (For interpretation of the references to color in this figure legend, the reader is referred to the web version of this article.)

Secondary analyses did not show significant differences between groups with a history of violence (NPV and PSY-V) and age-matched HC while controlling for the diagnosis of psychosis for any model or between psychosis patients (PSY-V and PSY-NV) while controlling for DDD of antipsychotic medication. All models revealed significantly higher BAG among psychosis groups (PSY-V and PSY-NV) compared with HC while controlling for history of violence, with 2.8 years (*p <.01*, *d* = 0.49) on average. The results are summarised in Fig. 3 and Supplementary Table T6. The BAG scores were explained by the contribution of psychosis diagnosis rather than a history of violence (rightmost dark red line Fig. 3a and the rightmost dark blue line Fig. 3b).

**Fig. 4:**
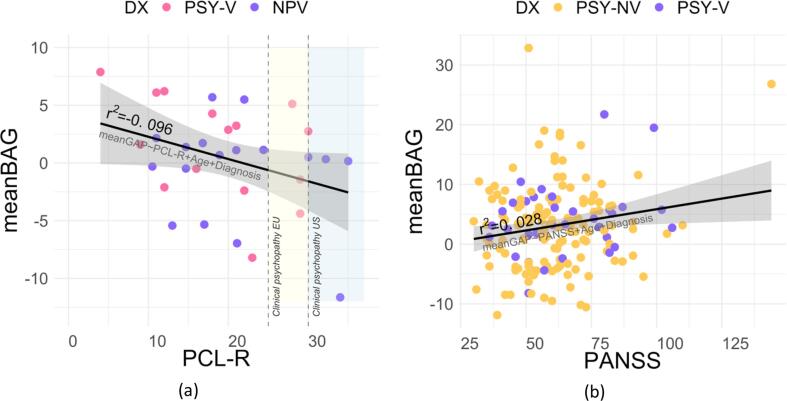
Relationship between PCL-R (a), total PANSS (b) scores and average of BAGs from 3 different models. The PCL-R scores increase with decreasing BAG, while PANSS total scores have an opposite pattern. Both relations are significant after controlling for diagnosis (DX) and participant’s age. Abbreviations: BAG – brain age gap, DX – diagnosis, NPV – violent offenders without psychosis, PSY-V – violent offenders with psychosis, PSY-NV – non-violent psychosis patients, PCL-R – Psychopathy Checklist-revised, PANSS – Positive and Negative Syndrome Scale.

PCL-R scores (Fig. 4a) were available for the subset of participants (n = 32) from NPV (n = 17) and PSY-V (n = 15) groups. There were no significant differences in PCL-R scores between NPV and PSY-V. The analysis revealed a significant negative association between PCL-R and average BAG while controlling for group and age *r^2^* = -0.096, *t* = 1.81, *d* = 1.17, *p* < 0.05. We repeated a similar analysis for PANSS total scores (Fig. 4b), with the data available for the participants from PSY-V (n = 34) and PSY-NV (n = 137) groups (total n = 171). The results showed a positive association between PANSS total score and mean BAG, *r^2^* = 0.0280, *t* = 2.22, *d* = 1.12, *p* < 0.05. Data for antipsychotic medication was available for 157 out of 176 participants from PSY-V and PSY-NV groups. The results revealed a positive association between antipsychotic medication and mean BAG, *r^2^* = 0.0236, *t* = 1.94, *d* = 0.91, *p* < 0.05. IQ scores did not significantly contribute to the model (*r^2^* = -0.057, *t* = 1.38, *d* = 0.45, *p* < 0.08). The results for associations for individual models are provided in the Supplementary Table T7.

The cross validation on the full set of HC is presented in Supplementary Table T8 and the complete list of fit results is presented in Table 3.

## 4 Discussion

In this study, we applied three different machine learning models based on structural MRI to estimate neurobiological age. Our results revealed several new insights into the interplay of violence, psychosis, and psychopathy based on neuroimaging predicted brain-age. First, we found a higher brain age gap (BAG) in the PSY-V and PSY-NV when compared with HC, but no significant differences between the PSY-V and PSY-NV, or between NPV and HC. Second, total PANSS scores in psychotic individuals with and without a history of violence were associated with higher BAG. Third, psychopathy scores (measured with PCL-R) in individuals with a history of violence with and without psychosis were negatively associated with BAG. The implications of these findings are discussed in detail below.

Our study revealed the BAG to be 4.9 years higher in the PSY-V and 2.7 years higher in the PSY-NV when compared to HC. These estimates are in line with our hypotheses and with previous reports on T1-based brain-age prediction in psychosis spectrum disorders, where increased BAG has been demonstrated throughout the course of illness, in individuals with high risk for psychosis (1.7 years) (Koutsouleris et al., 2014), first episode psychosis (2.6 years) (Kolenic et al., 2018) as well as in chronic schizophrenia disorder (up to 5.5 years) (Koutsouleris et al., 2014, Nenadic et al., 2017, Rokicki et al., 2021, Schnack et al., 2016). The observed stepwise trend of higher BAG in PSY-V (4.9 years versus 2.7 years in PSY-NV) may be due several causes, not necessarily exclusively linked to accelerated brain ageing, given that variations in brain-age can reflect developmental differences which show lifetime stability (Vidal-Pineiro et al., 2021).

Further, in the present sample, we did not find significant differences in the neuroanatomically predicted brain-age between the PSY-V and PSY-NV groups. Hence, our results showing higher BAG in the PSY-V when compared with HC may indicate that what we captured here was a cumulative load of progressive brain deficits associated with psychosis and linked to inherent disease mechanisms as well as secondary factors including lifestyle, medication and symptom severity. Thus, the contribution from brain structural abnormalities associated with a history of violence appears to be minor. Indeed, our supplementary analyses in the violence groups (PSY-V and NPV) and the psychosis groups (PSY-V and PSY-NV) versus HC revealed much larger case-control differences for the BAG due to psychosis (2.8 years) than due to violence (non-significant results). Additionally, we found a positive association between symptom severity in the psychosis groups (measured as total PANSS score) and BAG. This finding indicates a significant impact of psychosis symptom load on the predicted brain-age and adds to the accumulating evidence of increased BAG as a vulnerability marker of disease severity (Koutsouleris et al., 2014). The contribution of disease related factors to brain ageing in severe mental disorders aligns with previous studies in schizophrenia, which found significant associations between BAG and clinical variables including global assessment of functioning scale and PANSS (Kaufmann et al., 2019, Schnack et al., 2016). Moreover, we also found a positive association between DDD of antipsychotic medication and BAG in the psychosis groups, thus strengthening the hypothesis of iatrogenic contributions to brain ageing.

On the other hand, we can hypothesise that the observed pattern of higher BAG in PSY-V than PSY-NV compared with HC may point in the direction of more profoundly disrupted developmental trajectories during the critical time window in childhood and adolescence in this subgroup of patients. Indeed, it has been hypothesised that the neurodevelopmental abnormalities present in schizophrenia during the formative years may lead to deficient emotion processing and regulation as well as impaired integration and transfer of emotional input to higher cognitive brain regions and result in disruptive, aggressive behaviour (Hodgins, 2017, Hodgins and Klein, 2017). Indeed, widespread neuroanatomical deficits in schizophrenia patients with a history of violence compared with non-violent schizophrenia patients have been demonstrated, with most consistent neuroimaging findings showing volumetric reductions in orbitofrontal and anterior cingulate cortex (Fjellvang et al., 2018, Hoptman et al., 2014, Kumari et al., 2009). Additionally, a recent DTI-study from our group has reported a brain-wide pattern of reduced white matter integrity in psychosis patients with a history of violence when compared with non-violent psychosis patients (Tesli et al., 2021) in a subject sample overlapping with the current study.

One can speculate that higher brain-age in psychosis patients with a history of violence may be a product of an intricate interplay of the neurodevelopmental component, disorder-related processes, as well as the complex aggression phenotype (comorbidity with antisocial personality disorder and psychopathy). Given the inherent heterogeneity of violent and aggressive behaviour, it may be the case that we were not able to capture its unique influence on the neuroanatomical brain-age, despite operationalisation of violence according to the MacArthur criteria (Monahan et al., 2000), which unambiguously delineates the phenotype of interest to severe cases of trait violence. However, the significant associations between brain-age and psychopathy scores indicate that psychopathy traits may serve as a more specific proxy for the impact of antisocial behaviour on brain-age. Indeed, psychopathy scores in combination with anterior cingulate cortex reactivity (Aharoni et al., 2013) and age at release (Steele et al., 2015) have been shown to predict re-arrest among adult offenders. In our study, we found a similar level of psychopathy scores in the violent offenders with and without psychosis. These scores, in turn, were negatively associated with BAGs. This apparent reverted pattern of brain ageing effect associated with psychopathy traits may be understood in terms of a maturation delay linked to aberrant neurodevelopment and manifested by a deviant behavioural and cognitive profile (De Brito et al., 2021). Indeed, psychopathy is a syndrome characterised by widespread structural abnormalities in multiple brain networks (Johanson et al., 2019) involved in attention (Larson et al., 2013), emotional responsiveness (Viding and McCrory, 2019) as well as reinforcement-based decision making (De Brito et al., 2013). As demonstrated in a preliminary report by (Kiehl et al., 2018) inclusion of brain-age measures together with PCL-R scores and other sociodemographic variables improves prediction of re-offending, and outperforms the use of chronological age. Further, in this study, reduced grey matter volumes of inferior frontal and anterior temporal regions were the strongest predictors of brain-age (Kiehl et al., 2018). In our study, one of the most important structural features that contributed to the brain age prediction was thickness of the superior frontal cortex. The structural deficits in the superior-frontal regions have been shown to be involved in impulse control (Hu et al., 2016) as well as reward-based learning (Costa et al., 2016). Given the direction of the association (higher psychopathy score – lower brain-age), we can speculate that the driving process here may be linked to brain maturity delay manifested by volumetric and thickness abnormalities*,* as supported by imaging literature in youth samples exhibiting disruptive behaviour problems and callous-unemotional traits (De Brito et al., 2009, Yang et al., 2015).

The current findings should be interpreted in light of several limitations related to the design as well as sample characteristics. First, the application of a cross-sectional design to study brain-age makes any inferences regarding the incremental contribution of disease related processes versus the presence of inherent neurodevelopmental components associated with accelerated ageing challenging to address, particularly in light of the complex violence phenotype. Secondly, in similarity to other studies investigating brain-age in severe mental disorders, we were unable to differentiate between specific disease mechanisms from the impact of early life influences, lifestyle factors, and medication. Regarding possible residual confounding, we did not control the analyses for illicit substance or alcohol use. As individuals in both violence groups were institutionalised at the time of data collection (high security prison and psychiatry wards) they did not have, at least in theory, access to illicit substances or alcohol. However, given the impact of lifestyle factors on brain-age (Bittner et al., 2021), previous substance abuse might have affected the obtained results, particularly in the violent offenders with psychosis. Third, we had a limited number of subjects in the PSY-V and NPV groups. Nevertheless, the size of our violent psychosis group matches previous imaging studies investigating violence in schizophrenia (Del Bene et al., 2016). Moreover, to ensure that our findings were robust to overfitting we applied three different brain-age models and the results based on these three models converged (Supplementary Table T1). Fourth, not all the individuals in the violent psychosis group had the endurance to undergo the whole clinical protocol and we lacked PCL-R data for several participants. Thus, caution should be exercised when interpreting the results on the association between psychopathy scores and BAG.

Another important issue which merits attention is related to the obtained prediction accuracy. While the model fit was comparable to other studies which used FreeSurfer atlas based parcellation features (Beheshti et al., 2022), the performance was poorer compared to voxel-wise *T1w* based BAG studies (Bashyam et al., 2020, Beheshti et al., 2018, Beheshti et al., 2020). It can be speculated that this difference may partly be explained by the application of voxel-wise features versus summary statistics based on FreeSurfer atlases and parcellation procedures. Additionally, we cannot exclude the possibility that the differences in analysis pipelines and algorithms used may have contributed to the obtained estimates. Finally, accuracy of age prediction has been shown to depend on sample characteristics, including age range and sample size (de Lange et al., 2022).

Finally, while feature importance patterns showed a high agreement between models, some variance present in the obtained patterns could be explained by the difference in how each algorithm builds its prediction. XGBoost proceeds iteratively, with new trees that predict the residuals or errors of prior trees are combined with previous trees to make the final prediction. On the other hand, random forest is an ensemble of decision trees that builds multiple decision trees and averages them to obtain a more accurate and stable prediction. Importantly, random forest adds some randomness when building individual trees, i.e., uses only a subsample of available features, thus increasing robustness and decreasing chances of overfitting. Hence, more features are involved in brain age prediction when applying random forest compared to XGBoost. Also, as a consequence, the performance of random forest was poorer as compared with XGBoost models.

## 5 Conclusions

In summary, we report higher BAG in individuals with psychosis (with and without a history of violence) when compared with HC. Additionally, positive associations between psychosis symptoms scores and BAG, albeit no significant differences in BAG between violent and non-violent psychosis group suggest larger impact of symptom load than trait violence on brain-age. Further, negative associations between psychopathy scores and BAG in violent individuals (with and without psychosis) may indicate neuromaturation delay in individuals high on psychopathy traits. As structural, functional and diffusion measures convey different information related to brain health and ageing, future studies should combine larger datasets with multi-modal brain features to map neural age in violence and psychosis with higher accuracy. Additionally, given the complex interplay between structural brain development and phenotypic trajectories of violence, psychosis and psychopathy, future research should employ prospective longitudinal design to be able to disentangle contribution of disease specific and neurodevelopmental factors. In the long-term perspective, neuroimaging-based estimation of brain-age in individuals with a history violence and psychopathy traits may enhance our understanding of neurobiological mechanisms at work.

## Declaration of Competing Interest

The authors report no financial relationships with commercial interest, other than dr. Andreassen who received speakeŕs honorarium from Lundbeck and Sunovion, and is a consultant for HealthLytix.

## Data Availability

The data that has been used is confidential.
