# Supplementary material for: The age of violence: Mapping brain age in psychosis and psychopathy
Source: Neuroimage Clin. 2022 Sep 6;36:103181. doi: 10.1016/j.nicl.2022.103181 (PMC9474919; doi:10.1016/j.nicl.2022.103181)
Supplement: Supplementary data 1 [file mmc1.docx]

## Supplementary materials

##### M1. Participants: inclusion criteria

MRI data was available from 837 male participants. Among these, 52 were excluded due to insufficient T1w image quality, and 3 additional individuals were excluded due to spurious abnormal brain findings. Hence, the final sample comprised 782 individuals. All participants were recruited from the greater Oslo region as part of four studies: the Thematically Organized Psychosis study (TOP), the STROKEMRI study, The Youth TOP study (uTOP), and The Forensic Psychiatry study (sTOP).

1. The Forensic Psychiatry Study. Data for violent offenders with and without psychosis largely overlap with several publication from our research group, including (Bell et al., 2022; Storvestre et al., 2019; Tesli et al., 2021; Tesli et al., 2020; Tesli et al., 2019).
2. The Thematically Organized Psychosis study (TOP). Data for non-violent psychosis patients and healthy controls have been used in multiple studies across research groups from the TOP project, including large meta-analyses such as ENIGMA studies (Hibar et al., 2016; van Erp et al., 2016; van Erp et al., 2018).
3. The Youth TOP study (uTOP). Data for young participants with psychosis (<18 years) used in the current study may partly overlap with several studies from uTOP, including recent publications by (Morch-Johnsen et al., 2021; Smelror et al., 2021; Wedervang-Resell et al., 2020).
4. The StrokeMRI study. Data for elderly healthy controls from this study have been used in several studies including brain age prediction research ((Richard et al., 2018; Richard et al., 2020; Sanders et al., 2021).

The inclusion criteria for participants in the TOP study (non-violent psychosis group, PSY-NV) were following: a diagnosis of psychosis spectrum disorder based on the DSM-IV criteria, age between 18 and 65 years, no history of severe head injury or other disorders affecting the central nervous system, and an IQ > 70. The participants were referred to the study by their clinicians from local hospitals. Healthy control subjects (HC) were randomly selected from the Norwegian national population registry (<http://www.ssb.no/en>) and invited to participate. Upon inclusion in the TOP study, the HC were screened with the Primary Care Evaluation of Mental Disorders (PRIME-MD) to confirm no history of psychiatric disorder. Younger participants (aged between 12-18 years) were included from the Youth-TOP study sample. The inclusion criteria for these participants were similar to the TOP study, a psychosis diagnosis was based on DSM-IV criteria using the Norwegian version of the Schedule for Affective Disorders and Schizophrenia for School Aged Children (6–18 years) present and lifetime version (K-SADS-PL) (Kaufman et al., 1997). All participants and their guardians provided written informed consent to participate in the study.

The HC in the STROKEMRI study were recruited through local newspapers and social media. The inclusion criteria for this group were age at or above 18, no history of stroke, dementia, or other neurological and psychiatric disorders, no alcohol or substance abuse or intake of medications significantly affecting the nervous system.

The inclusion of younger (Youth-TOP) and older (STROKEMRI) participants enabled us to train our machine-learning algorithms on a wide range of ages (12-92 years). Hence, the brain prediction algorithms were able to estimate both higher and lower brain-age compared with chronological age in patient groups (15.1-71.0 years).

The inclusion criteria for participants in the sTOP study (violent offenders with psychosis (PSY-V), and non-psychotic violent offenders (NPV)) were age between 18 and 70 years, IQ>65, absence of head trauma leading to loss of consciousness and no current or previous somatic illness that might have affected brain morphology. The NPV group consisted of incarcerated persons serving a preventive detention sentence, which is the most severe sanction according to the Norwegian penal law and is imposed in cases of particularly serious crimes involving interpersonal violence. The sanction can be prolonged as long as the offender is considered to pose a significant risk to others, which may involve a life-long imprisonment. As of December 2021, a total of 150 persons were serving a preventive detention sentence in Norway.

The inclusion of younger (Youth-TOP) and older (STROKEMRI) participants enabled us to train our machine-learning algorithms on a wide range of ages (12-92 years). Hence, the brain prediction algorithms were able to estimate both higher and lower brain-age compared with chronological age in patient groups (15.1-71.0 years).

M2. The complete list of all brain structures used for brain age prediction

**Cortical structures:** 1. Left bankssts 2. Left caudalanteriorcingulate 3. Left caudalmiddle-frontal 4. Left cuneus 5. Left entorhinal 6. Left fusiform 7. Left inferiorparietal 8. Left inferiortemporal 9. Left isthmuscingulate 10. Left lateraloccipital 11. Left lingual 12. Left lateralorbitofrontal 13. Left medialorbitofrontal 14. Left middletemporal 15. Left parahippocampal 16. Left paracentral 17. Left parsopercularis 18. Left parsorbitalis 19. Left parstriangularis 20. Left pericalcarine 21. Left postcentral 22. Left posteriorcingulate 23. Left precentral 24. Left precuneus 25. Left rostralanteriorcingulate 26. Left superiorfrontal 27. Left rostralmiddlefrontal 28. Left superiorparietal 29. Left superiortemporal 30. Left supramarginal 31. Left frontalpole 32. Left temporalpole 33. Left transversetemporal 34. Left in- sula 35. Right bankssts 36. Right caudalanteriorcingulate 37. Right caudalmiddlefrontal 38. Right cuneus 39. Right entorhinal 40. Right fusiform 41. Right inferiorparietal 42. Right inferiortemporal 43. Right isthmuscingulate 44. Right lateraloccipital 45. Right lateralorbitofrontal 46. Right lingual 47. Right medialorbitofrontal 48. Right middletemporal 49. Right parahippocampal 50. Right paracentral 51. Right parsopercularis 52. Right parsorbitalis 53. Right parstriangularis 54. Right pericalcarine 55. Right postcentral 56. Right posteriorcingulate 57. Right precentral 58. Right precuneus 59. Right rostralanteriorcingulate 60. Right rostralmiddlefrontal 61. Right superiorfrontal 62. Right superiorparietal 63. Right superiortemporal 64. Right supramarginal 65. Right frontalpole 66. Right temporalpole 67. Right transversetemporal 68. Right insula

**Subcortical structures**: 1. Left Lateral Ventricle 2. Left Inf Lat Vent 3. Left Cerebellum White Matter 4. Left Cerebellum Cortex 5. Left Thalamus 6. Left Caudate 7. Left Putamen 8. Left Pallidum 9. X3rd Ventricle 10. X4th Ventricle 11. Brain Stem 12. Left Hippocampus 13. Left Amygdala 14. CSF 15. Left Accumbens area 16. Left VentralDC 17. Left vessel 18. Left choroid plexus 19. Right Lateral Ventricle 20. Right Inf Lat Vent 21. Right Cerebellum White Matter 22. Right Cerebellum Cortex 23. Right Thalamus 24. Right Caudate 25. Right Putamen 26. Right Pallidum 27. Right Hippocampus 28. Right Amygdala 29. Right Accumbens area 30. Right VentralDC 31. Right Ventral 32. Right choroid plexus 33. X5th ventricle volume 34. WM hypointensities 35. Left WM hypointensities 36. Right WM hypointensities 37. non WM hypointensities 38. Left non WM hypointensities 39. Right non WM hypointensities 40. Optic Chiasm 41. CC Posterior 42. CC Mid Posterior 43. CC Central 44. CC Mid Anterior 45. CC Anterior

**Hippocampal subfields and amygdala nuclei**: 1. Lateral nucleus 2. Basal nucleus 3. Accessory 4. Basal nucleus 5. Anterior amygdaloid area AAA 6. Central nucleus 7. Medial nucleus 8. Cortical nucleus 9. Corticoamygdaloid transition 10. Paralaminar nucleus 11. Hippocampal tail subiculum.body 12. CA1 body 13. Subiculum head 14. hippocampal fissure 15. presubiculum head 16. CA1 head 17. Presubiculum body 18. Parasubiculum 19. molecular layer HP head 20. molecular layer HP body 21. GC ML DG head 22. CA3 body 23. GC ML DG body 24. CA4 head 25. CA4 body 26. Fimbria 27. CA3 head 28. HATA 29. Whole hippocampal body 30. Whole hippocampal head

| 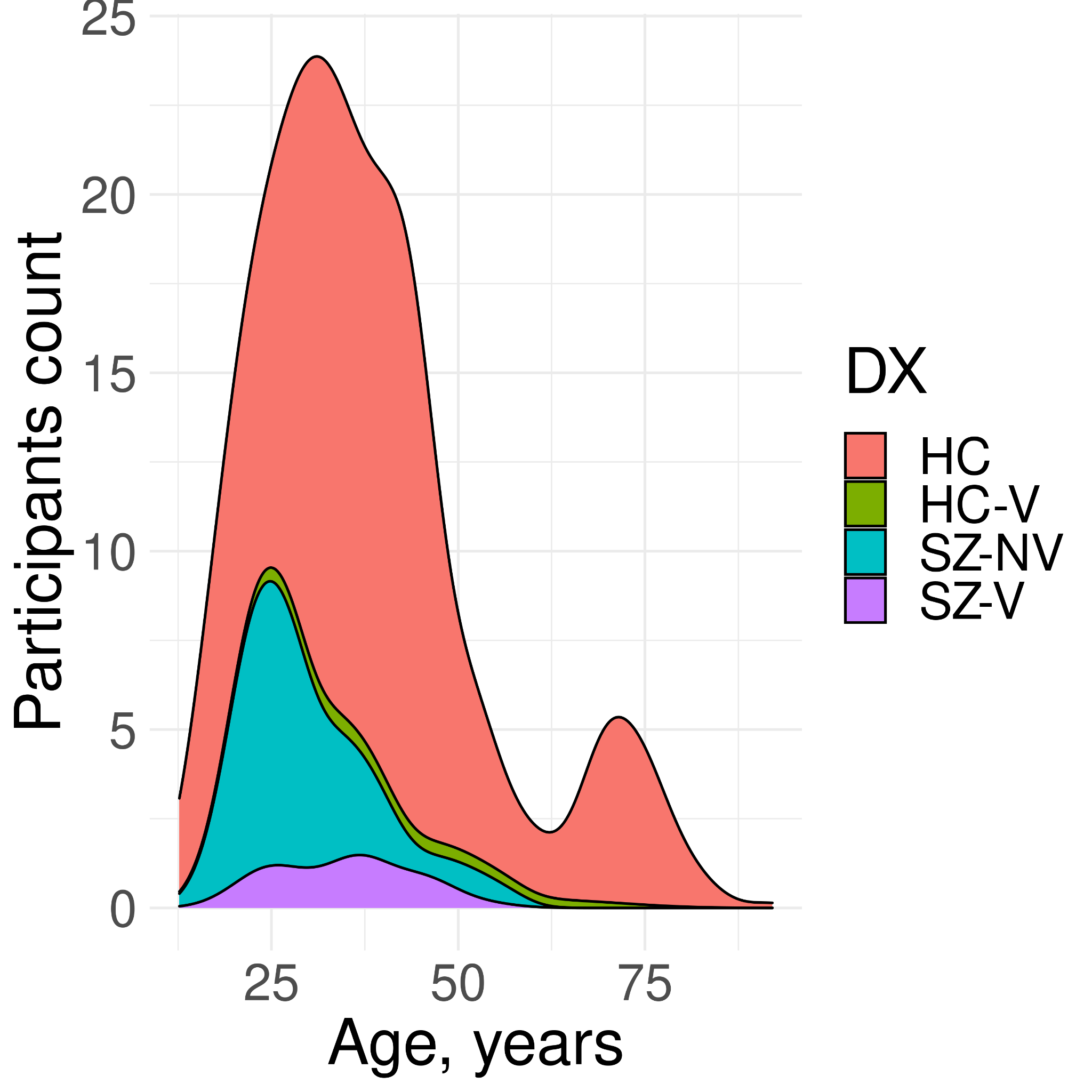  **(a)** | **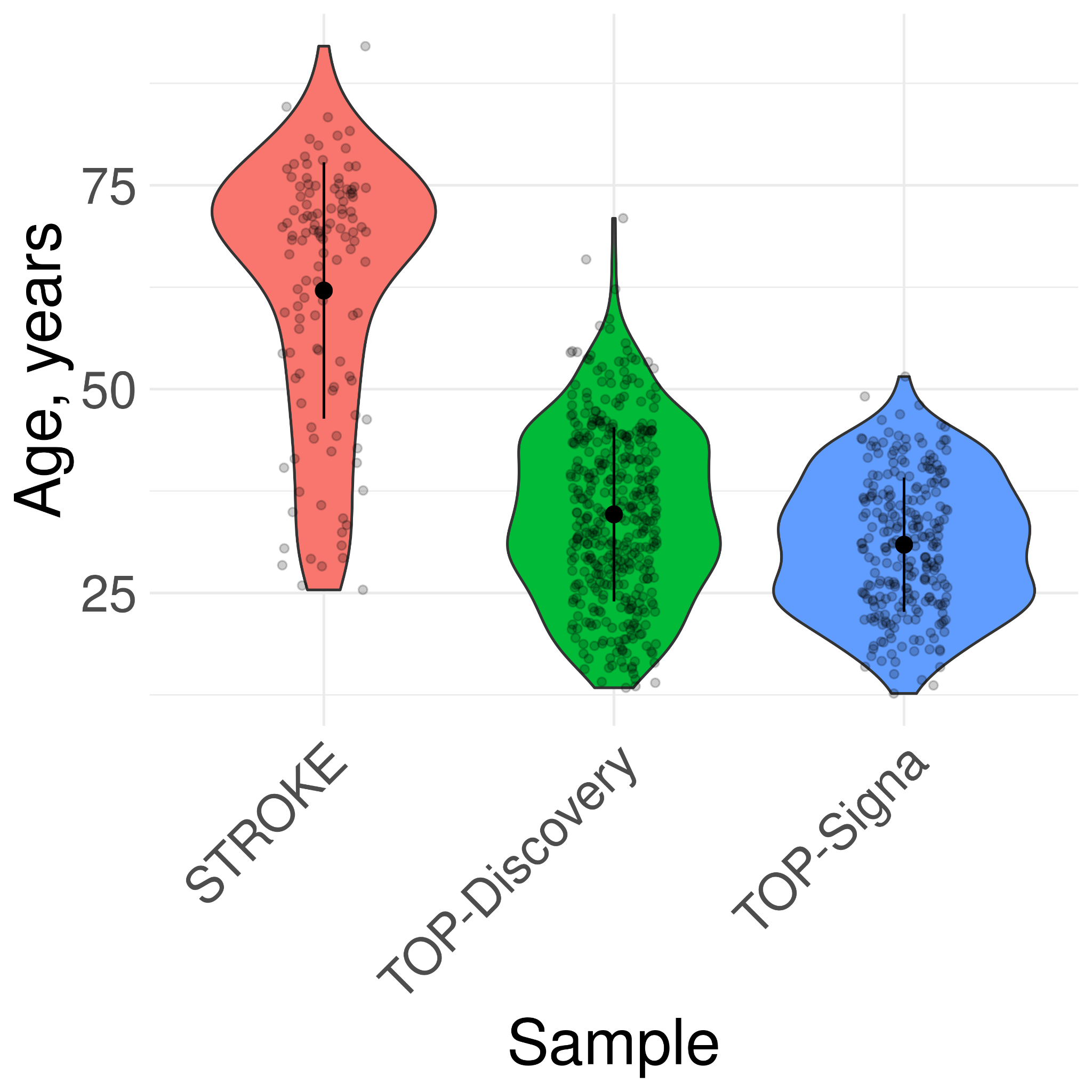**  **(b)** |
| --- | --- |
| **Figure S1.** Participant demographics grouped by diagnosis (a) and sample (b). | |


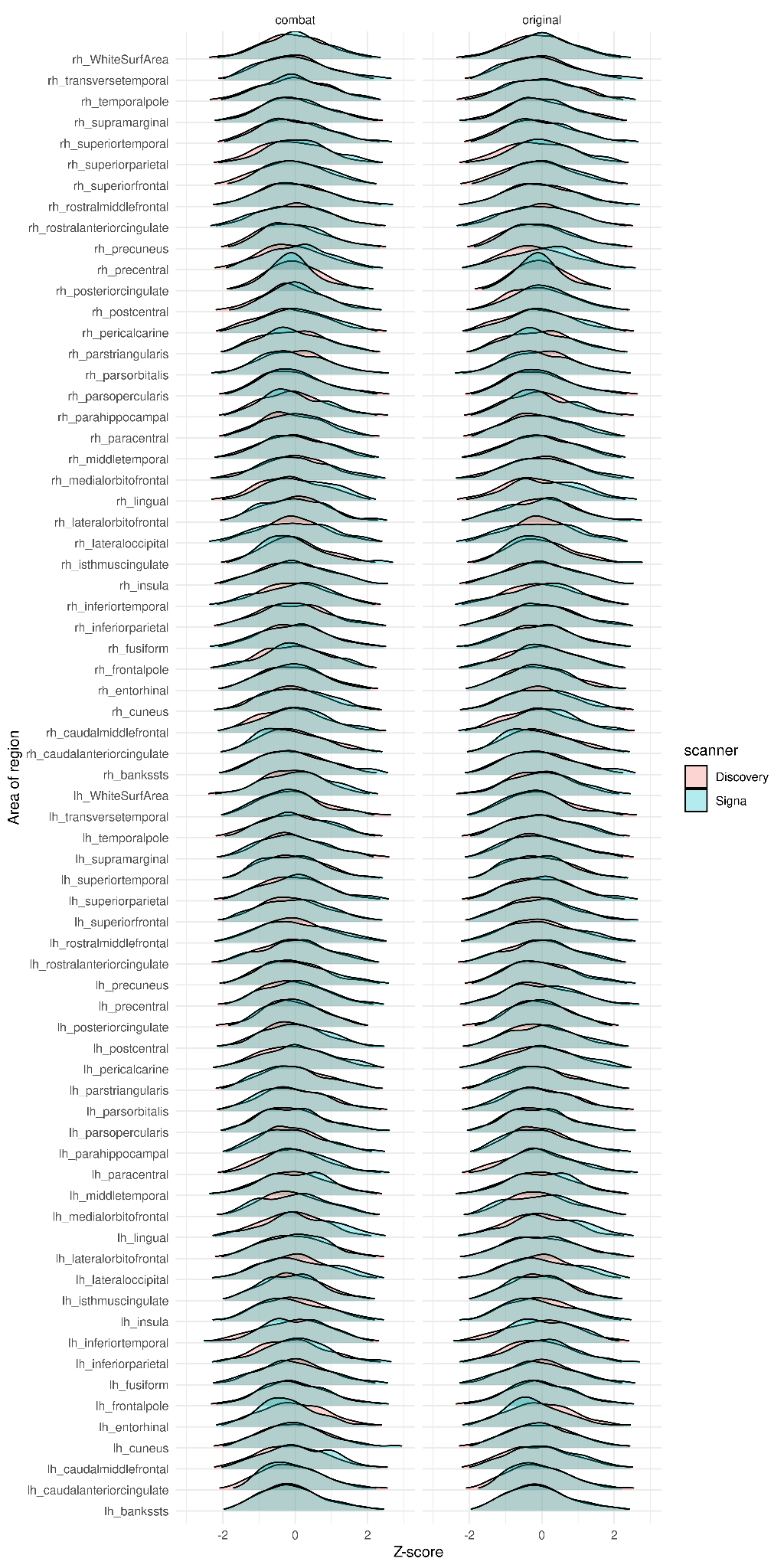


**Figure S2.** Area harmonization for scanner effects after (1st column) and before (2nd column) combat application. Area values were converted to Z-values within each region to enhance visualisation.


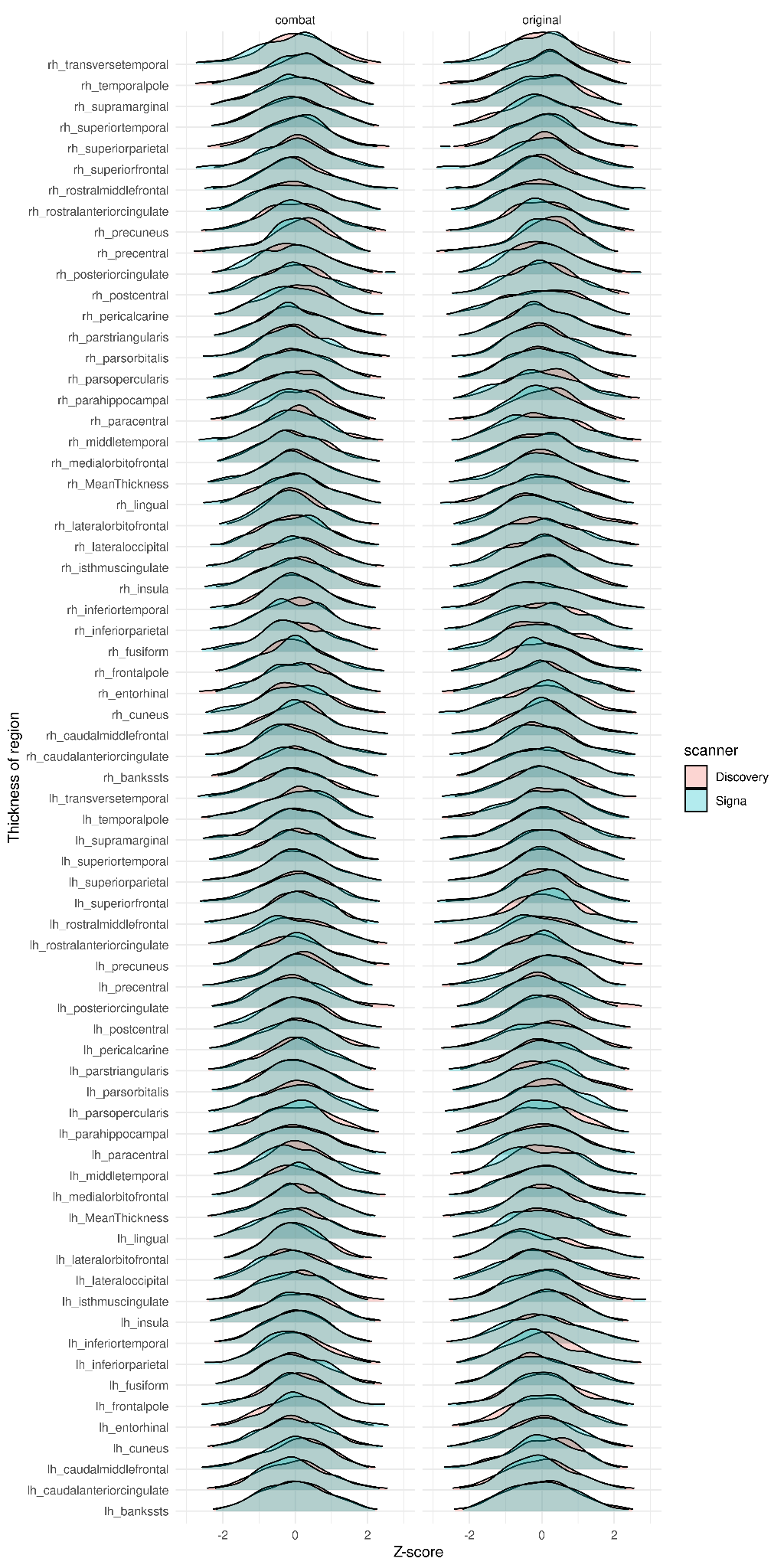


**Figure S3.** Thickness harmonization for scanner effects after (1st column) and before (2nd column) combat application. Thickness values were converted to Z-values within each region to enhance visualisation.


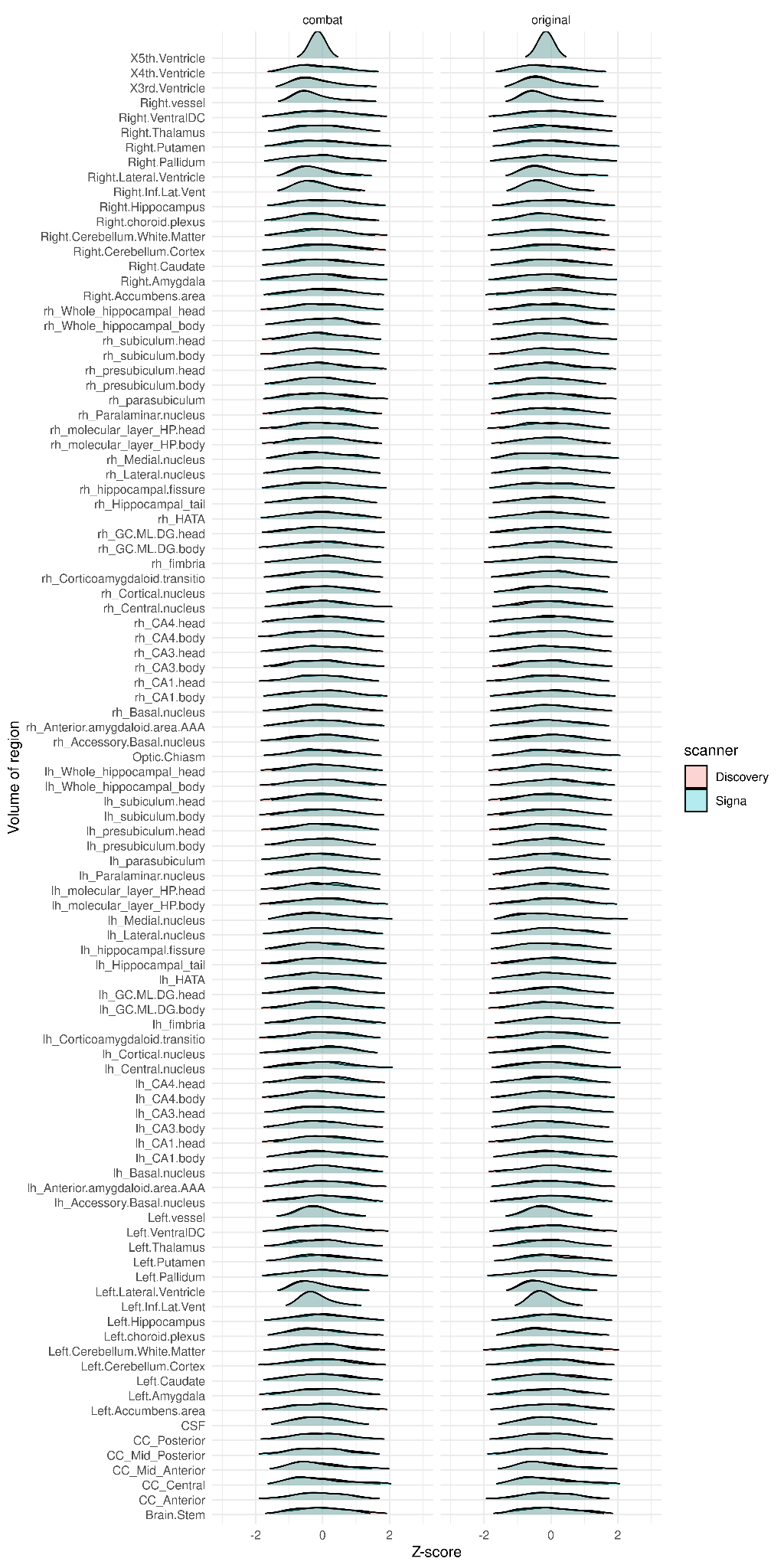


**Figure S4.** Volume harmonization for scanner effects after (1st column) and before (2nd column) combat application. Volumes were converted to Z-values within each region to enhance visualisation.


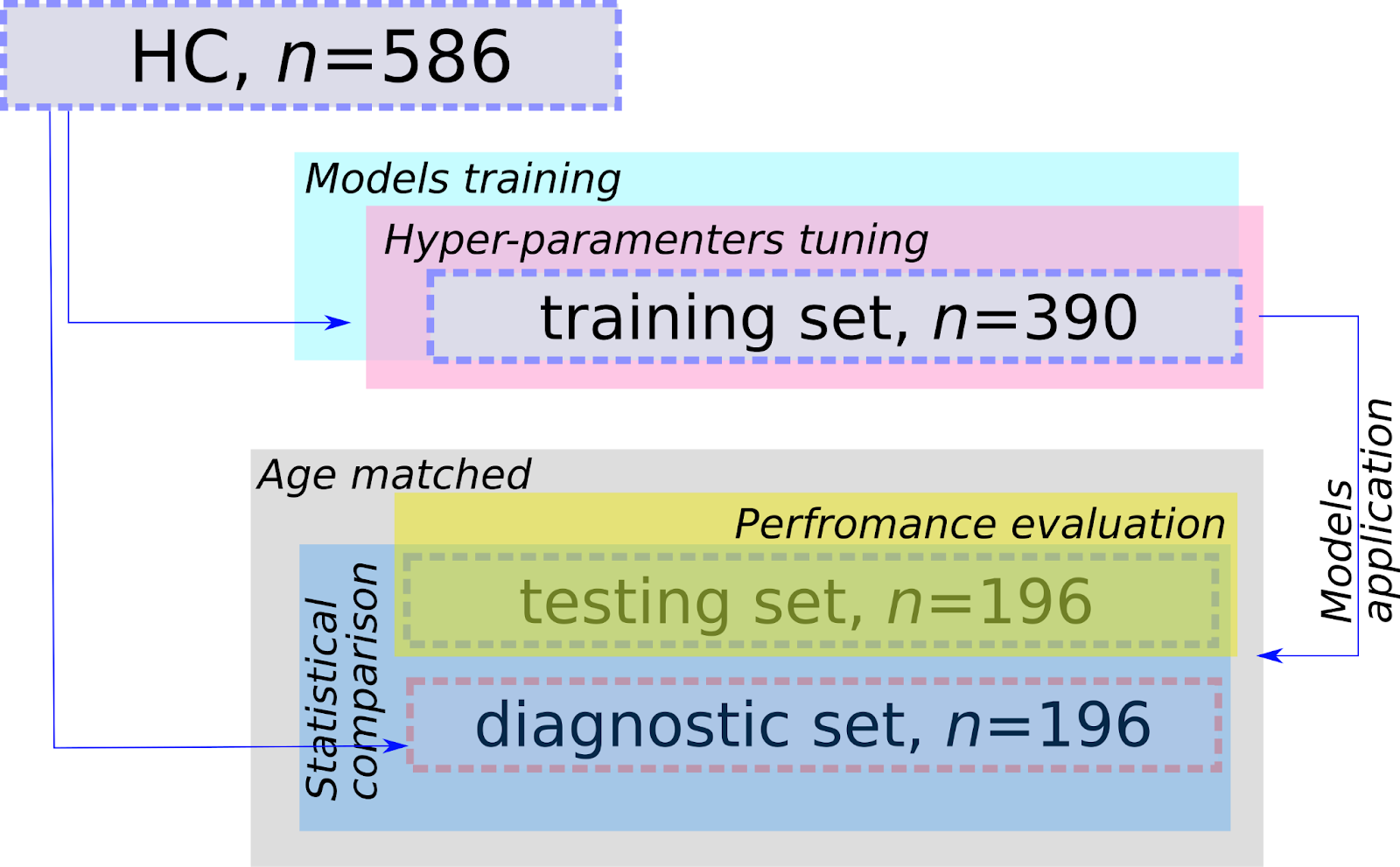


**Figure S5.** Study workflow.

| 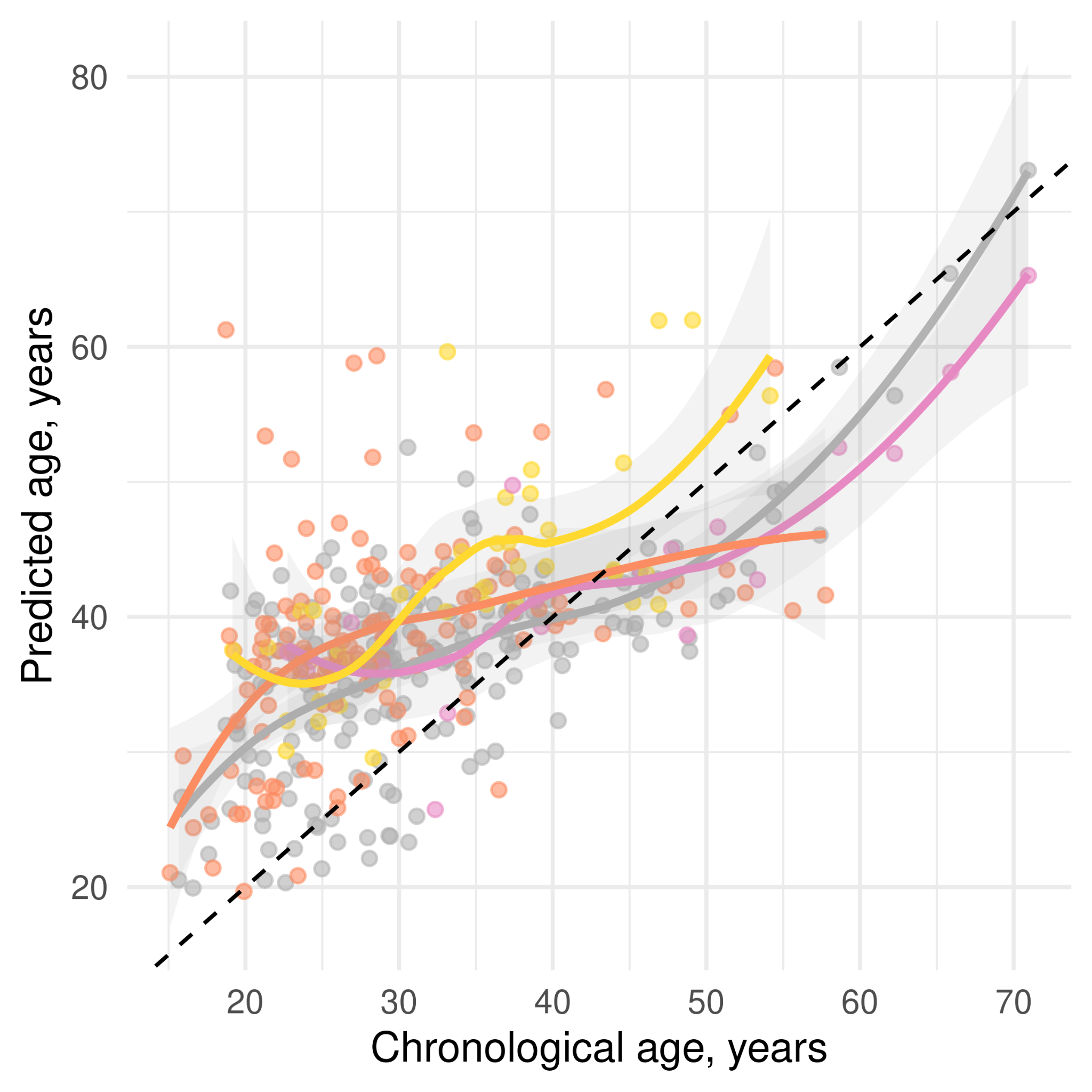  (a) | 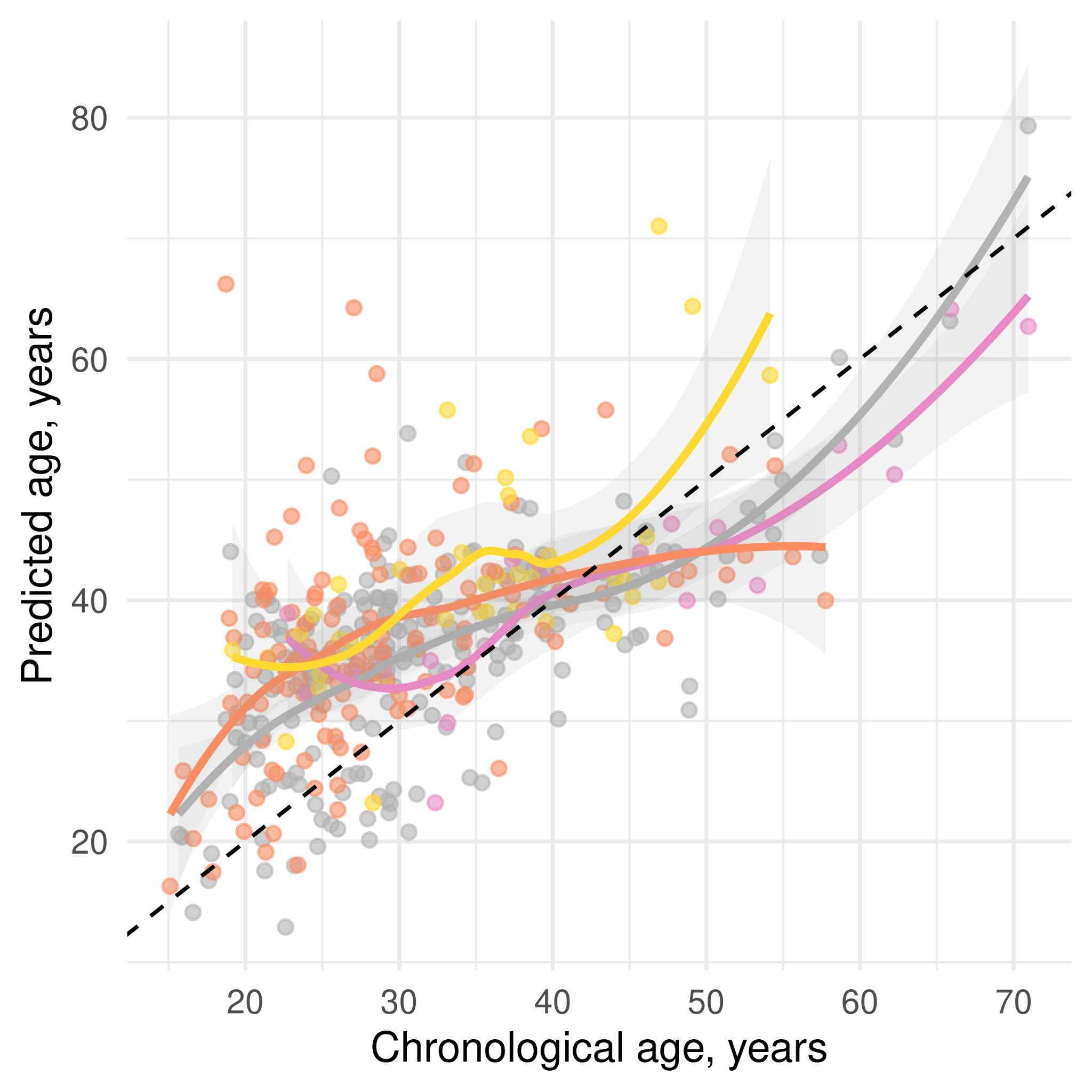  (b) |
| --- | --- |
| 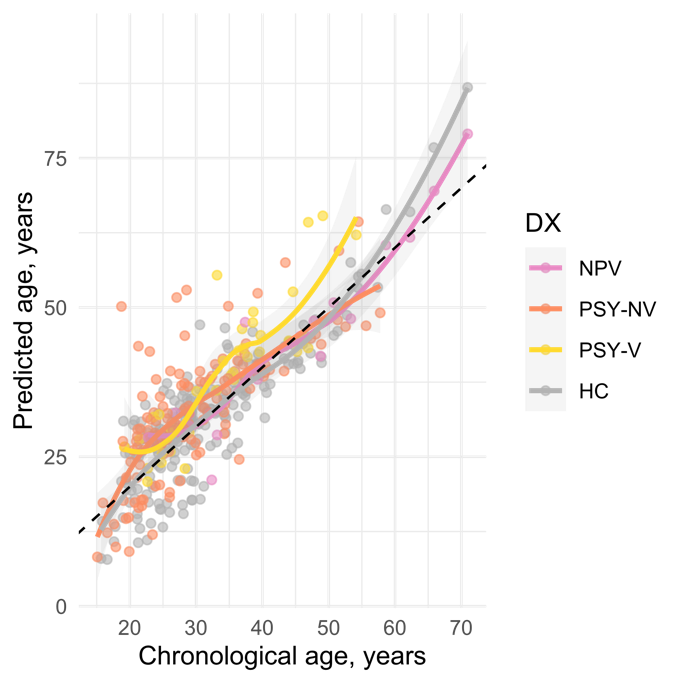  (c) | 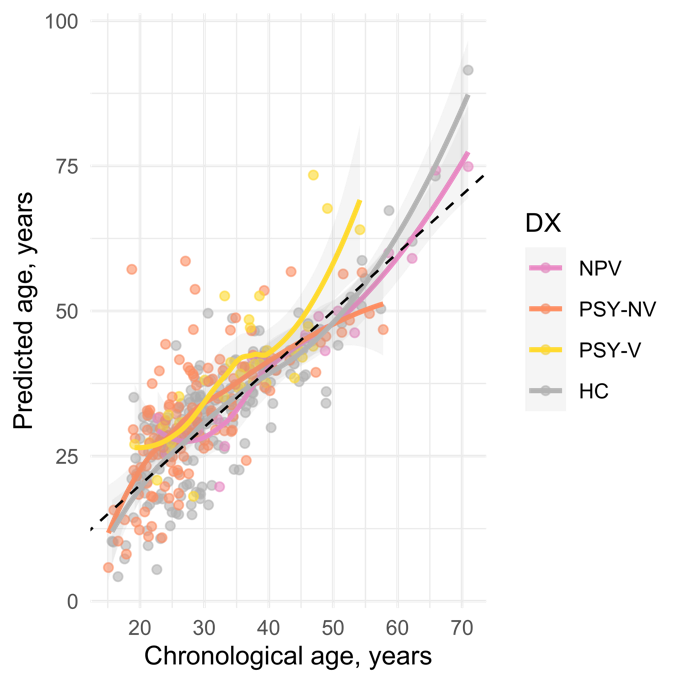  (d) |

**Figure S6.** Association between chronological and predicted age based on all modalities. Color points and lines represent different patient groups. Solid black line represents the ideal fit, dashed black line represents the fit based on HC, on which the model was trained. Based on random forests (a) and xgboost with optimised parameters (b) before and after residualising for age (c) and (d) correspondingly. Abbreviations: HC – healthy controls, NPV - violent offenders without psychosis; PSY-V - violent offenders with psychosis; PSY-NV - non-violent psychosis patients.

| 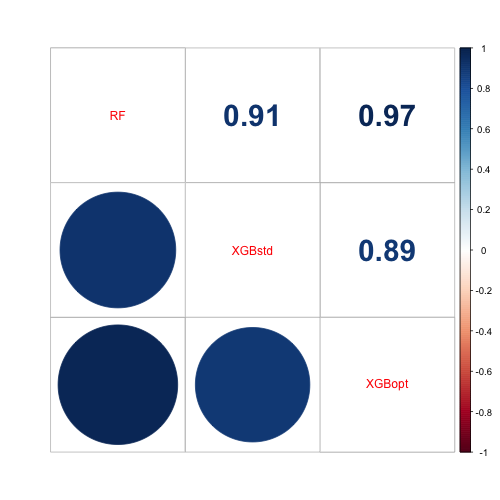   (a) | 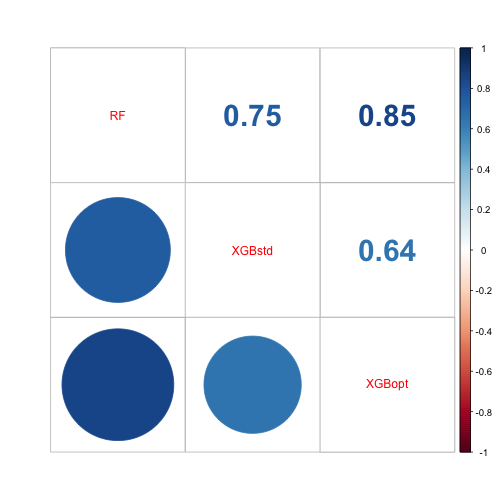  (b) |
| --- | --- |
| 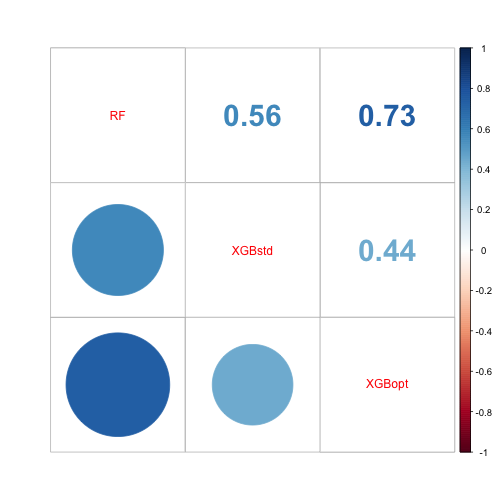  (c) | 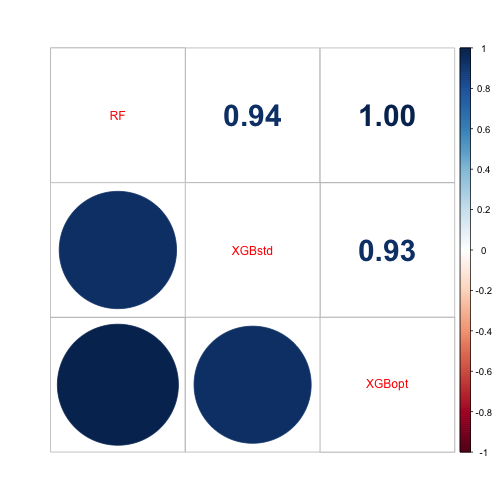  (d) |

**Figure S7.** Pearson’s correlation between all (a), thickness only (b), area only (c) and volume only (d) feature importance values for brain age prediction. Feature importance was measured as an increase in MSE for random forest model and as a gain for xgboost models. All correlations were significant at *p*<0.05 after Bonferroni adjusting for 12 comparisons, 4 modalities x 3 models.

| 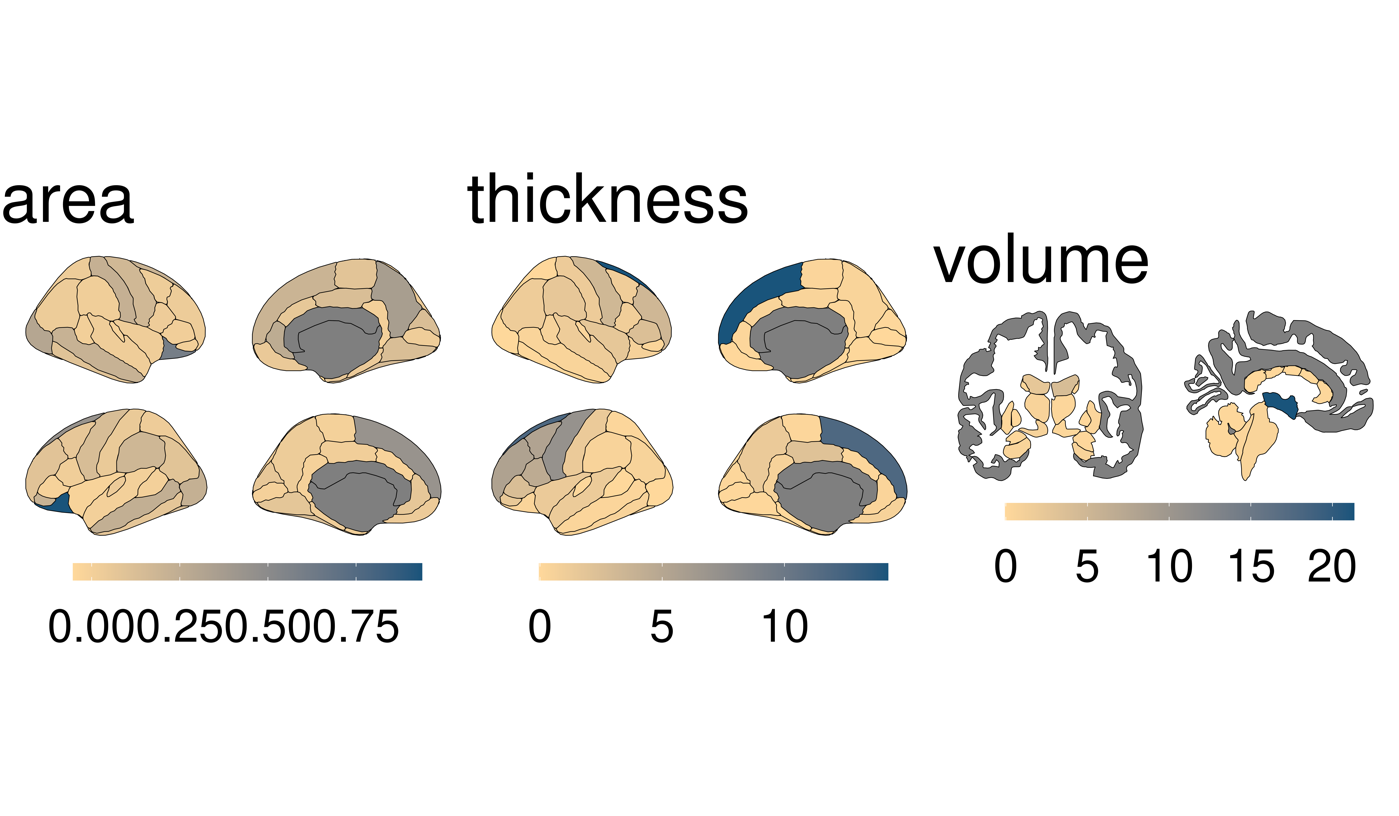  (a) |
| --- |
| 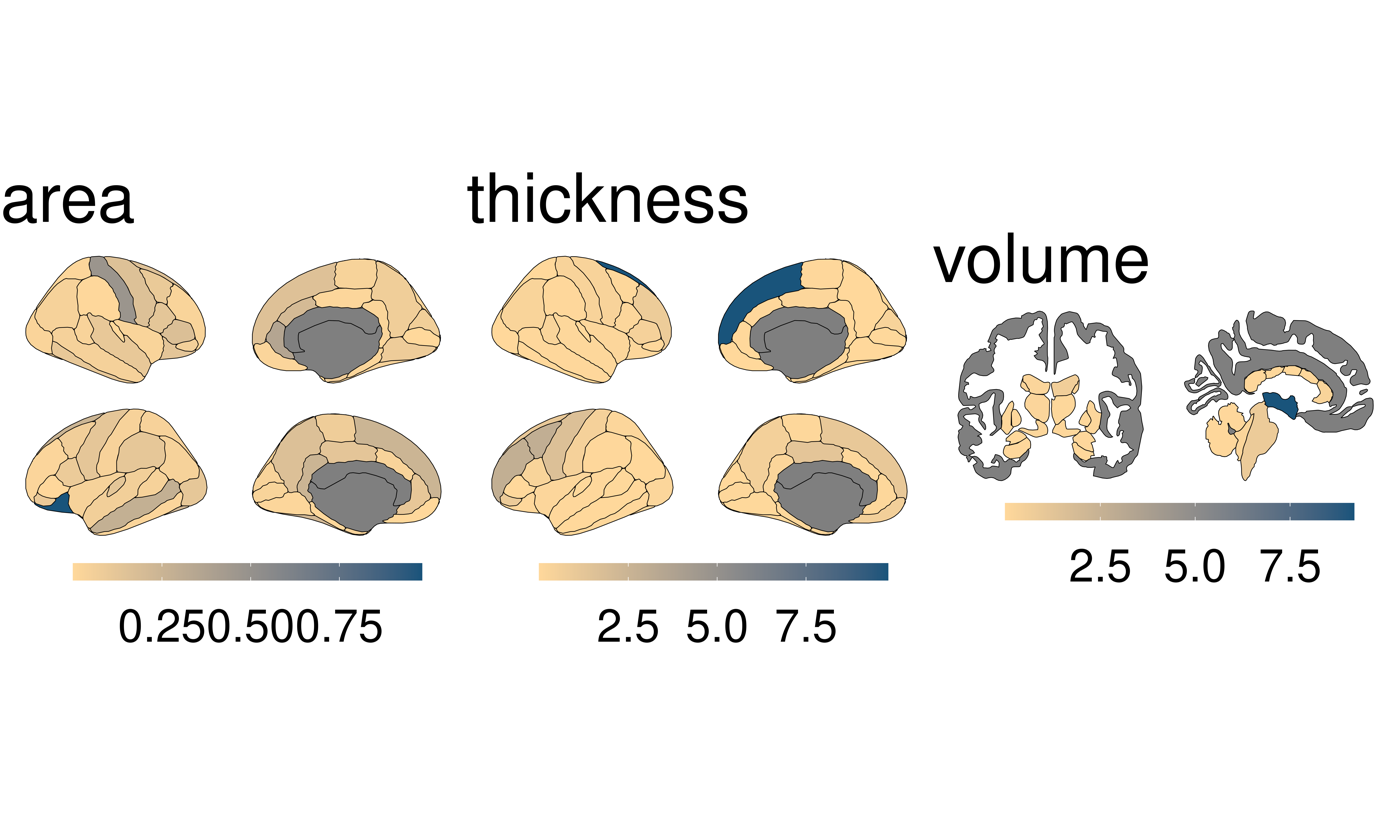  (b) |

**Figure S8.** Feature importance measured as increase in MSE (left) shown as a colormap overlaid on the brain for random forests for random forests model (a) and as a gain for xgboost model with parameters optimisation (b).

|  | **HC** | | | **Patients/incarcerated** | | |
| --- | --- | --- | --- | --- | --- | --- |
|  | RF | XGB_opt | XGB_std | RF | XGB_opt | XGB_std |
| RF | 1.00 | 0.95 | 0.92 | 1.00 | 0.94 | 0.92 |
| XGB_opt |  | 1.00 | 0.91 |  | 1.00 | 0.92 |
| XGB_std |  |  | 1.00 |  |  | 1.00 |
| **Table T1.** Correlation of brain age prediction by different methods, RF – random forests, XGB-opt – xgboost with parameters optimisation, XGB-std – xgboost with default parameters. All correlations p<0.05. | | | | | | |

| **Hemisphere** | **Structure** | **Type** | **Gain** | **Cover** | **Frequency** |
| --- | --- | --- | --- | --- | --- |
| bi | WM hypointensities | volume | 17.91 | 0.0132 | 0.0065 |
| bi | X3rd Ventricle | volume | 13.81 | 0.0156 | 0.0081 |
| rh | superiorfrontal | thickness | 5.60 | 0.0095 | 0.0053 |
| lh | superiorfrontal | thickness | 3.78 | 0.0063 | 0.0032 |
| rh | Accumbens | volume | 3.78 | 0.0129 | 0.0073 |
| rh | Lateral Ventricle | volume | 3.41 | 0.0030 | 0.0032 |
| lh | parstriangularis | thickness | 3.21 | 0.0080 | 0.0057 |
| rh | fimbria | volume | 2.70 | 0.0070 | 0.0053 |
| rh | precentral | thickness | 2.66 | 0.0096 | 0.0061 |
| rh | MeanThickness | thickness | 2.22 | 0.0038 | 0.0016 |
| lh | precentral | thickness | 2.12 | 0.0063 | 0.0061 |
| lh | bankssts | thickness | 1.55 | 0.0062 | 0.0348 |
| lh | precuneus | thickness | 1.35 | 0.0037 | 0.0036 |
| bi | Brain.Stem | volume | 1.24 | 0.0133 | 0.0097 |
| lh | caudalmiddlefrontal | thickness | 1.10 | 0.0049 | 0.0162 |
| lh | posteriorcingulate | thickness | 1.01 | 0.0031 | 0.0041 |
| lh | superiorfrontal | area | 0.76 | 0.0091 | 0.0061 |
| rh | superiortemporal | thickness | 0.71 | 0.0041 | 0.0041 |
| lh | Lateral Ventricle | volume | 0.62 | 0.0021 | 0.0020 |
| rh | Hippocampal tail | volume | 0.62 | 0.0045 | 0.0045 |
| **Table T2.** Feature importance for the xgboost model with standard parameters. Only the top 20 most informative features are shown. Abbreviations: lh – left hemisphere, rh – right hemisphere, bi – bilateral. | | | | | |

| **Hemisphere** | **Structure** | **Type** | **Gain** | **Cover** | **Frequency** |
| --- | --- | --- | --- | --- | --- |
| bi | WM hypointensities | volume | 26.73 | 0.0090 | 0.0078 |
| rh | superiorfrontal | thickness | 9.80 | 0.0026 | 0.0028 |
| bi | X3rd Ventricle | volume | 9.18 | 0.0032 | 0.0040 |
| rh | MeanThickness | thickness | 5.91 | 0.0060 | 0.0045 |
| lh | MeanThickness | thickness | 4.36 | 0.0065 | 0.0048 |
| lh | caudalmiddlefrontal | thickness | 2.95 | 0.0032 | 0.0033 |
| lh | rostralmiddlefrontal | thickness | 2.87 | 0.0021 | 0.0028 |
| rh | fimbria | volume | 2.74 | 0.0053 | 0.0049 |
| rh | Accumbens | area | 2.33 | 0.0024 | 0.0024 |
| lh | fimbria | volume | 2.32 | 0.0036 | 0.0041 |
| lh | precentral | thickness | 1.64 | 0.0021 | 0.0025 |
| lh | posteriorcingulate | thickness | 1.28 | 0.0023 | 0.0035 |
| lh | superiorfrontal | thickness | 1.08 | 0.0031 | 0.0031 |
| lh | lateralorbitofrontal | area | 0.98 | 0.0146 | 0.0112 |
| lh | parsorbitalis | thickness | 0.90 | 0.0031 | 0.0040 |
| bi | brain stem | volume | 0.86 | 0.0038 | 0.0046 |
| rh | rostralmiddlefrontal | thickness | 0.86 | 0.0027 | 0.0028 |
| rh | Lateral Ventricle | volume | 0.63 | 0.0041 | 0.0039 |
| lh | medialorbitofrontal | thickness | 0.60 | 0.0054 | 0.0053 |
| lh | precuneus | thickness | 0.58 | 0.0009 | 0.0018 |
| **Table T3.** Feature importance for the xgboost model with parameter optimisation. Only the top 20 most informative features are shown. Abbreviations: lh – left hemisphere, rh – right hemisphere, bi – bilateral. | | | | | |

| Hemisphere | Structure | Type | %IncMSE | IncNodePurity |
| --- | --- | --- | --- | --- |
| bi | WM hypointensities | volume | 59.91 | 18,706.64 |
| bi | X3rd Ventricle | volume | 21.31 | 9,500.47 |
| rh | MeanThickness | thickness | 16.57 | 5,413.03 |
| rh | superiorfrontal | thickness | 14.23 | 5,684.45 |
| lh | superiorfrontal | thickness | 11.90 | 7,062.50 |
| lh | precentral | thickness | 7.20 | 2,415.22 |
| lh | fimbria | volume | 7.14 | 2,659.39 |
| rh | fimbria | volume | 6.90 | 1,991.76 |
| lh | MeanThickness | thickness | 6.79 | 2,969.29 |
| rh | Accumbens | volume | 5.53 | 2,106.13 |
| lh | rostralmiddlefrontal | thickness | 5.53 | 3,257.48 |
| lh | caudalmiddlefrontal | thickness | 5.35 | 2,668.54 |
| lh | parsopercularis | thickness | 4.53 | 2,311.25 |
| rh | Lateral Ventricle | volume | 4.11 | 1,595.28 |
| rh | rostralmiddlefrontal | thickness | 3.29 | 1,299.21 |
| rh | precentral | thickness | 3.25 | 1,149.38 |
| lh | parstriangularis | thickness | 2.60 | 846.18 |
| lh | Lateral Ventricle | volume | 2.53 | 891.37 |
| rh | parstriangularis | thickness | 2.46 | 954.50 |
| lh | Accumbens | volume | 2.20 | 779.11 |
| **Table T4.** Feature importance for the random forests model. Only the top 20 most informative features are shown. Abbreviations: lh – left hemisphere, rh – right hemisphere, bi – bilateral. | | | | |

| **DX** | **N** | **mean(Age)** | **sd(Age)** | **min(Age)** | **max(Age)** | **p-value** |
| --- | --- | --- | --- | --- | --- | --- |
| HC/NPV | 20/20 | 42.8 | 14.0 | 23.5 | 70.9 | 0.94 |
|  |  | 42.4 | 14.4 | 22.7 | 71.0 |  |
| HC/PSY-NV | 138/138 | 29.4 | 8.3 | 15.7 | 57.4 | 0.67 |
|  |  | 29.0 | 8.7 | 15.1 | 57.8 |  |
| HC/PSY-V | 38/38 | 35.0 | 8.4 | 19.5 | 54.4 | 0.89 |
|  |  | 34.7 | 8.9 | 19.2 | 54.1 |  |
| **Table T5.** Participants’ demographics for group comparison. Subjects were matched by the means of matchIt package using nearest neighbour matching with 1:1 ratio and logistic regression distance. Along with the number of subjects in each group (N=20/20 meaning that we have 20 HC and 20 NPV participants), mean age, minimal age and maximal age in each group and p-value from two group comparisons are shown. There are no significant differences in age in any of the comparisons. Abbreviations: HC – healthy controls, NPV - violent offenders without psychosis; PSY-V – violent offenders with psychosis; PSY-NV – non-violent psychosis patients. | | | | | | |

|  | BAG diff, years | Cd | t-value | p_ADJUSTED_ |  |
| --- | --- | --- | --- | --- | --- |
|  | **non-Psychotic/Psychotic (n=176/176)** | | | | |
| *RF* | 3.05 | 0.58 | 4.23 | 1.20e-03 | ** |
| *XGB_std* | 2.41 | 0.44 | 3.18 | 5.60e-03 | ** |
| *XGB_opt* | 2.74 | 0.45 | 3.30 | 5.60e-03 | ** |
|  | **non-Violent/Violent (n=58/58)** | | | | |
| *RF* | 0.39 | 0.09 | 0.29 | 1.00e+00 |  |
| *XGB_std* | 0.67 | 0.14 | 0.42 | 1.00e+00 |  |
| *XGB_opt* | -0.25 | -0.05 | -0.15 | 1.00e+00 |  |
|  | **PSY-NV/PSY-V (n=35/34) controlling for DDD** | | | | |
| *RF* | 0.03 | 0.02 | 0.03 | 1.00e+00 |  |
| *XGB_std* | 0.29 | 0.15 | 0.26 | 0.96e+00 |  |
| *XGB_opt* | -0.11 | -0.05 | -0.09 | 1.00e+00 |  |
| **Table T6.** Mean group differences between age matched HC with violence controlled for psychosis and psychosis controlled for violence and between PSY-NV and PSY-V controlling for age and defined daily dose (DDD) of antipsychotic medication. BAG was additionally controlled for age within each comparison, using linear models. P-values were adjusted for 3 models x 2 conditions = 6 comparisons using false discovery rate (FDR). Cd stands for Cohen’s d. T-values and p-values were calculated with PALM using permutation modelling. | | | | | |

| Model | R_Pearson_ | r^2^ | Cohen’s d | p_uncorrected_ |
| --- | --- | --- | --- | --- |
|  | PCL-R association (n=32) | | | |
| Random forest | -0.2605 | -0.0679 | 0.9743 | 0.073 |
| xgboost-opt | -0.2740 | -0.0751 | 1.0490 | 0.0571 |
| xgboost-std | -0.3188 | -0.1016 | 1.1874 | 0.0367 |
| **Mean BAG** | **-0.3094** | **-0.0957** | **1.167** | **0.0383** |
|  | PANSS association (n=171) | | | |
| Random forest | 0.1651 | 0.0273 | 1.1030 | 0.0160 |
| xgboost-opt | 0.1297 | 0.0168 | 0.8637 | 0.0436 |
| xgboost-std | 0.1912 | 0.0366 | 1.2887 | 0.0068 |
| **Mean BAG** | **0.1672** | **0.0280** | **1.1204** | **0.0136** |
|  | IQ association (n=316) | | | |
| Random forest | -0.0820 | -0.0067 | 0.4883 | 0.0617 |
| xgboost-opt | -0.0393 | -0.0015 | 0.2310 | 0.2383 |
| xgboost-std | -0.0991 | -0.0098 | 0.5877 | 0.0317 |
| **Mean BAG** | **-0.0753** | **-0.0057** | **0.4465** | **0.0796** |

**Table T7.** Results for the associations for PANSS, PCL-R and IQ for individual models.

|  |  | **XGB-opt** | | | **XGB-std** | | | **Random Forest** | | |
| --- | --- | --- | --- | --- | --- | --- | --- | --- | --- | --- |
| *FOLD* | *n* | *r^2^* | *mae* | *rmse* | *r^2^* | *mae* | *rmse* | *r^2^* | *mae* | *rmse* |
| 1 | 44 | 0.86 | 5.9 | 7.3 | 0.89 | 4.9 | 6.1 | 0.86 | 5.6 | 7.2 |
| 2 | 63 | 0.85 | 5.7 | 6.9 | 0.89 | 5.0 | 6.2 | 0.88 | 5.2 | 6.4 |
| 3 | 68 | 0.80 | 6.2 | 7.6 | 0.81 | 6.0 | 7.6 | 0.79 | 6.7 | 8.3 |
| 4 | 74 | 0.84 | 4.8 | 6.2 | 0.86 | 4.7 | 5.8 | 0.83 | 5.2 | 6.5 |
| 5 | 61 | 0.74 | 6.1 | 7.2 | 0.75 | 5.7 | 7.1 | 0.68 | 6.3 | 8.0 |
| 6 | 63 | 0.81 | 5.2 | 6.6 | 0.84 | 5.0 | 6.1 | 0.84 | 4.9 | 6.0 |
| 7 | 47 | 0.88 | 4.6 | 5.9 | 0.88 | 4.7 | 6.1 | 0.87 | 4.8 | 6.1 |
| 8 | 58 | 0.82 | 5.8 | 7.3 | 0.80 | 6.3 | 7.8 | 0.81 | 6.4 | 7.6 |
| 9 | 45 | 0.78 | 5.1 | 6.4 | 0.78 | 5.2 | 6.5 | 0.74 | 5.2 | 6.8 |
| 10 | 63 | 0.78 | 5.1 | 6.1 | 0.77 | 5.1 | 6.3 | 0.78 | 5.0 | 6.2 |
| **TOTAL** | **586** | **0.82** | **5.5** | **6.8** | **0.83** | **5.3** | **6.6** | **0.82** | **5.6** | **7.0** |

**Table T8.** 10-fold cross validation within the healthy control sample (n=568). Results are presented before the brain age bias removal.

**References**

Bell, C., Tesli, N., Gurholt, T.P., Rokicki, J., Hjell, G., Fischer-Vieler, T., Melle, I., Agartz, I., Andreassen, O.A., Rasmussen, K., Johansen, R., Friestad, C., Haukvik, U.K., 2022. Associations between amygdala nuclei volumes, psychosis, psychopathy, and violent offending. Psychiatry Res Neuroimaging 319, 111416.

Hibar, D.P., Westlye, L.T., van Erp, T.G., Rasmussen, J., Leonardo, C.D., Faskowitz, J., Haukvik, U.K., Hartberg, C.B., Doan, N.T., Agartz, I., Dale, A.M., Gruber, O., Kramer, B., Trost, S., Liberg, B., Abe, C., Ekman, C.J., Ingvar, M., Landen, M., Fears, S.C., Freimer, N.B., Bearden, C.E., Costa Rica/Colombia Consortium for Genetic Investigation of Bipolar, E., Sprooten, E., Glahn, D.C., Pearlson, G.D., Emsell, L., Kenney, J., Scanlon, C., McDonald, C., Cannon, D.M., Almeida, J., Versace, A., Caseras, X., Lawrence, N.S., Phillips, M.L., Dima, D., Delvecchio, G., Frangou, S., Satterthwaite, T.D., Wolf, D., Houenou, J., Henry, C., Malt, U.F., Boen, E., Elvsashagen, T., Young, A.H., Lloyd, A.J., Goodwin, G.M., Mackay, C.E., Bourne, C., Bilderbeck, A., Abramovic, L., Boks, M.P., van Haren, N.E., Ophoff, R.A., Kahn, R.S., Bauer, M., Pfennig, A., Alda, M., Hajek, T., Mwangi, B., Soares, J.C., Nickson, T., Dimitrova, R., Sussmann, J.E., Hagenaars, S., Whalley, H.C., McIntosh, A.M., Thompson, P.M., Andreassen, O.A., 2016. Subcortical volumetric abnormalities in bipolar disorder. Mol Psychiatry 21(12), 1710-1716.

Kaufman, J., Birmaher, B., Brent, D., Rao, U., Flynn, C., Moreci, P., Williamson, D., Ryan, N., 1997. Schedule for Affective Disorders and Schizophrenia for School-Age Children-Present and Lifetime Version (K-SADS-PL): initial reliability and validity data. J Am Acad Child Adolesc Psychiatry 36(7), 980-988.

Morch-Johnsen, L., Smelror, R.E., Andreou, D., Barth, C., Johannessen, C., Wedervang-Resell, K., Wortinger, L.A., Diaz, R., Victoria, G., Ueland, T., Andreassen, O.A., Myhre, A.M., Rund, B.R., Ulloa, R.E., Agartz, I., 2021. Negative Symptom Domains Are Associated With Verbal Learning in Adolescents With Early Onset Psychosis. Front Psychiatry 12, 825681.

Richard, G., Kolskar, K., Sanders, A.M., Kaufmann, T., Petersen, A., Doan, N.T., Monereo Sanchez, J., Alnaes, D., Ulrichsen, K.M., Dorum, E.S., Andreassen, O.A., Nordvik, J.E., Westlye, L.T., 2018. Assessing distinct patterns of cognitive aging using tissue-specific brain age prediction based on diffusion tensor imaging and brain morphometry. PeerJ 6, e5908.

Richard, G., Kolskar, K., Ulrichsen, K.M., Kaufmann, T., Alnaes, D., Sanders, A.M., Dorum, E.S., Monereo Sanchez, J., Petersen, A., Ihle-Hansen, H., Nordvik, J.E., Westlye, L.T., 2020. Brain age prediction in stroke patients: Highly reliable but limited sensitivity to cognitive performance and response to cognitive training. Neuroimage Clin 25, 102159.

Sanders, A.M., Richard, G., Kolskar, K., Ulrichsen, K.M., Kaufmann, T., Alnaes, D., Beck, D., Dorum, E.S., de Lange, A.G., Egil Nordvik, J., Westlye, L.T., 2021. Linking objective measures of physical activity and capability with brain structure in healthy community dwelling older adults. Neuroimage Clin 31, 102767.

Smelror, R.E., Johannessen, C., Wedervang-Resell, K., Jorgensen, K.N., Barth, C., Andreou, D., Ueland, T., Andreassen, O.A., Myhre, A.M., Rund, B.R., Agartz, I., 2021. Cognitive impairment profile in adolescent early-onset psychosis using the MATRICS Battery: Age and sex effects. Neuropsychology 35(3), 300-309.

Storvestre, G.B., Valnes, L.M., Jensen, A., Nerland, S., Tesli, N., Hymer, K.E., Rosaeg, C., Server, A., Ringen, P.A., Jacobsen, M., Andreassen, O.A., Agartz, I., Melle, I., Haukvik, U.K., 2019. A preliminary study of cortical morphology in schizophrenia patients with a history of violence. Psychiatry Res Neuroimaging 288, 29-36.

Tesli, N., Rokicki, J., Maximov, I.I., Bell, C., Hjell, G., Gurholt, T., Fischer-Vieler, T., Bang, N., Tesli, M., Westlye, L.T., Andreassen, O.A., Melle, I., Agartz, I., Rasmussen, K., Johansen, R., Friestad, C., Haukvik, U.K., 2021. White Matter Matters: Unraveling Violence in Psychosis and Psychopathy. Schizophrenia Bulletin Open 2(1).

Tesli, N., van der Meer, D., Rokicki, J., Storvestre, G., Rosaeg, C., Jensen, A., Hjell, G., Bell, C., Fischer-Vieler, T., Tesli, M., Andreassen, O.A., Melle, I., Agartz, I., Haukvik, U.K., 2020. Hippocampal subfield and amygdala nuclei volumes in schizophrenia patients with a history of violence. Eur Arch Psychiatry Clin Neurosci 270(6), 771-782.

Tesli, N., Westlye, L.T., Storvestre, G.B., Gurholt, T.P., Agartz, I., Melle, I., Andreassen, O.A., Haukvik, U.K., 2019. White matter microstructure in schizophrenia patients with a history of violence. Eur Arch Psychiatry Clin Neurosci.

van Erp, T.G., Hibar, D.P., Rasmussen, J.M., Glahn, D.C., Pearlson, G.D., Andreassen, O.A., Agartz, I., Westlye, L.T., Haukvik, U.K., Dale, A.M., Melle, I., Hartberg, C.B., Gruber, O., Kraemer, B., Zilles, D., Donohoe, G., Kelly, S., McDonald, C., Morris, D.W., Cannon, D.M., Corvin, A., Machielsen, M.W., Koenders, L., de Haan, L., Veltman, D.J., Satterthwaite, T.D., Wolf, D.H., Gur, R.C., Gur, R.E., Potkin, S.G., Mathalon, D.H., Mueller, B.A., Preda, A., Macciardi, F., Ehrlich, S., Walton, E., Hass, J., Calhoun, V.D., Bockholt, H.J., Sponheim, S.R., Shoemaker, J.M., van Haren, N.E., Hulshoff Pol, H.E., Ophoff, R.A., Kahn, R.S., Roiz-Santianez, R., Crespo-Facorro, B., Wang, L., Alpert, K.I., Jonsson, E.G., Dimitrova, R., Bois, C., Whalley, H.C., McIntosh, A.M., Lawrie, S.M., Hashimoto, R., Thompson, P.M., Turner, J.A., 2016. Subcortical brain volume abnormalities in 2028 individuals with schizophrenia and 2540 healthy controls via the ENIGMA consortium. Mol Psychiatry 21(4), 547-553.

van Erp, T.G.M., Walton, E., Hibar, D.P., Schmaal, L., Jiang, W., Glahn, D.C., Pearlson, G.D., Yao, N., Fukunaga, M., Hashimoto, R., Okada, N., Yamamori, H., Bustillo, J.R., Clark, V.P., Agartz, I., Mueller, B.A., Cahn, W., de Zwarte, S.M.C., Hulshoff Pol, H.E., Kahn, R.S., Ophoff, R.A., van Haren, N.E.M., Andreassen, O.A., Dale, A.M., Doan, N.T., Gurholt, T.P., Hartberg, C.B., Haukvik, U.K., Jorgensen, K.N., Lagerberg, T.V., Melle, I., Westlye, L.T., Gruber, O., Kraemer, B., Richter, A., Zilles, D., Calhoun, V.D., Crespo-Facorro, B., Roiz-Santianez, R., Tordesillas-Gutierrez, D., Loughland, C., Carr, V.J., Catts, S., Cropley, V.L., Fullerton, J.M., Green, M.J., Henskens, F.A., Jablensky, A., Lenroot, R.K., Mowry, B.J., Michie, P.T., Pantelis, C., Quide, Y., Schall, U., Scott, R.J., Cairns, M.J., Seal, M., Tooney, P.A., Rasser, P.E., Cooper, G., Shannon Weickert, C., Weickert, T.W., Morris, D.W., Hong, E., Kochunov, P., Beard, L.M., Gur, R.E., Gur, R.C., Satterthwaite, T.D., Wolf, D.H., Belger, A., Brown, G.G., Ford, J.M., Macciardi, F., Mathalon, D.H., O'Leary, D.S., Potkin, S.G., Preda, A., Voyvodic, J., Lim, K.O., McEwen, S., Yang, F., Tan, Y., Tan, S., Wang, Z., Fan, F., Chen, J., Xiang, H., Tang, S., Guo, H., Wan, P., Wei, D., Bockholt, H.J., Ehrlich, S., Wolthusen, R.P.F., King, M.D., Shoemaker, J.M., Sponheim, S.R., De Haan, L., Koenders, L., Machielsen, M.W., van Amelsvoort, T., Veltman, D.J., Assogna, F., Banaj, N., de Rossi, P., Iorio, M., Piras, F., Spalletta, G., McKenna, P.J., Pomarol-Clotet, E., Salvador, R., Corvin, A., Donohoe, G., Kelly, S., Whelan, C.D., Dickie, E.W., Rotenberg, D., Voineskos, A.N., Ciufolini, S., Radua, J., Dazzan, P., Murray, R., Reis Marques, T., Simmons, A., Borgwardt, S., Egloff, L., Harrisberger, F., Riecher-Rossler, A., Smieskova, R., Alpert, K.I., Wang, L., Jonsson, E.G., Koops, S., Sommer, I.E.C., Bertolino, A., Bonvino, A., Di Giorgio, A., Neilson, E., Mayer, A.R., Stephen, J.M., Kwon, J.S., Yun, J.Y., Cannon, D.M., McDonald, C., Lebedeva, I., Tomyshev, A.S., Akhadov, T., Kaleda, V., Fatouros-Bergman, H., Flyckt, L., Karolinska Schizophrenia, P., Busatto, G.F., Rosa, P.G.P., Serpa, M.H., Zanetti, M.V., Hoschl, C., Skoch, A., Spaniel, F., Tomecek, D., Hagenaars, S.P., McIntosh, A.M., Whalley, H.C., Lawrie, S.M., Knochel, C., Oertel-Knochel, V., Stablein, M., Howells, F.M., Stein, D.J., Temmingh, H.S., Uhlmann, A., Lopez-Jaramillo, C., Dima, D., McMahon, A., Faskowitz, J.I., Gutman, B.A., Jahanshad, N., Thompson, P.M., Turner, J.A., 2018. Cortical Brain Abnormalities in 4474 Individuals With Schizophrenia and 5098 Control Subjects via the Enhancing Neuro Imaging Genetics Through Meta Analysis (ENIGMA) Consortium. Biol Psychiatry 84(9), 644-654.

Wedervang-Resell, K., Ueland, T., Aukrust, P., Friis, S., Holven, K.B., C, H.J., Lekva, T., Lonning, V., Smelror, R.E., Szabo, A., Andreassen, O.A., Myhre, A.M., Agartz, I., 2020. Reduced levels of circulating adhesion molecules in adolescents with early-onset psychosis. NPJ Schizophr 6(1), 20.
